# Endophytic fungus, *Chaetomium globosum,* associated with marine green alga, a new source of Chrysin

**DOI:** 10.1038/s41598-020-72497-3

**Published:** 2020-10-30

**Authors:** Siya Kamat, Madhuree Kumari, Kuttuvan Valappil Sajna, C. Jayabaskaran

**Affiliations:** Department of Biochemistry, Indian Institute of Science, Bangalore, 560012 India

**Keywords:** Biochemistry, Biological techniques, Biotechnology, Cancer, Drug discovery, Microbiology, Plant sciences

## Abstract

The marine ecosystem is an extraordinary reserve of pharmaceutically important, bioactive compounds even in this “synthetic age”. Marine algae-associated endophytic fungi have gained prominence as an important source of bioactive compounds. This study was conducted on secondary metabolites of *Chaetomium globosum*-associated with marine green alga *Chaetomorpha media* from the Konkan coastline, India. Its ethyl acetate extract (CGEE) exhibited an IC_50_ value of 7.9 ± 0.1 µg/mL on MCF-7 cells. CGEE exhibited G2M phase cell cycle arrest, ROS production and MMP loss in MCF-7 cells. The myco-components in CGEE contributing to the cytotoxicity were found by Gas Chromatography/Mass Spectrometry analyses. Chrysin, a dihydroxyflavone was one of the forty-six myco-components which is commonly found in honey, propolis and passionflower extracts. The compound was isolated and characterized as fungal chrysin using HPLC, UV–Vis spectroscopy, LC–MS, IR and NMR analyses by comparing with standard chrysin. The purified compound exhibited an IC_50_ value of 49.0 ± 0.6 µM while that of standard chrysin was 48.5 ± 1.6 µM in MCF-7 cells. It induced apoptosis, G1 phase cell cycle arrest, MMP loss, and ROS production. This is the first report of chrysin from an alternative source with opportunities for yield enhancement.

## Introduction

Through centuries, indigenous cultures around the globe have used and developed natural strategies to treat a myriad of illnesses. These distinct natural non-nutrient compounds are secondary metabolites that have garnered a lot of attention in the scientific community^1,2^. Being target specific and less toxic to normal cells, they have unleashed new paradigms in the search for therapeutics^3^.

Flavonoids are a ubiquitous class of secondary metabolites, commonly found in flowers, fruits, and to a lesser extent in fungi^4^. It is well established through epidemiological, clinical, and animal studies that flavonoids and its derivatives display shielding effects against cancer by interacting with the nucleotide-binding sites of kinases, phospholipases, ATPase, lipooxygenases, cyclooxygenases, and phosphodiesterases^5^. They are also known to modulate apoptotic pathways via increasing levels of reactive oxygen species (ROS) and induction of DNA damage^4^.

**Figure 1:**
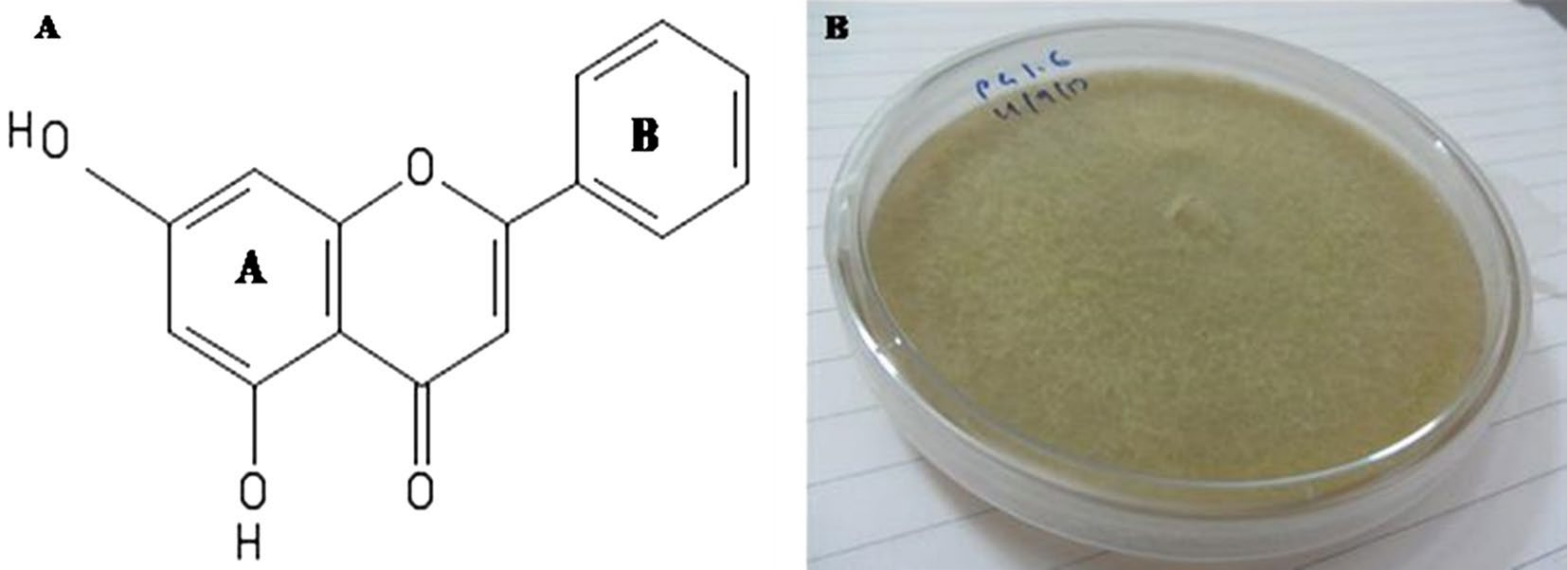
(**A**) Structure of Chrysin (5,7-dihydroxyflavone). (**B**) Seven days old endophytic fungus *Chaetomium globosum* (PG 1.6) cultured on potato dextrose agar.

Chrysin (5,7-dihydroxyflavone) is a flavone commonly found in propolis, honey, and passion fruit. Recent findings have also reported chrysin from *Radix Scutellariae* and *Pleurotus ostreatus*^6^. As seen in Fig. 1A, the structure of this compound is composed of an oxygen-bearing heterocyclic ring and two benzene rings (A and B). It holds double bonds and –OH groups in its A ring. Chrysin, unlike other flavonoids, lacks oxygenation in its B ring. Thus, it falls under the class of flavones. Derivatives of chrysin are mainly generated due to diversity in oxygenation, examples of which are apigenin, wogonin, baicalein, and oroxylin^7^.

Biological activity of chrysin is attributed to the deficiency of oxygenation in its rings B and C. Various reports on the biological significance of this compound have demonstrated that it possesses anti-cancer properties. The cytotoxic effects of chrysin were studied on human uveal melanoma cell lines and it was observed that chrysin (IC_50_ values, 30–100 µM), selectively induced apoptosis via the intrinsic pathway^8^. In a combination study with metformin on T47D breast cancer cells, suppression of hTERT and cyclin D1 levels was observed^9^. In breast cancer-induced mouse models, chrysin demonstrated antimetastatic activity as well as rescinded hypoxia-induced angiogenesis^10^. Its anti-angiogenic activity was reported to be associated with a reduction in levels of MMP-2 and MMP-9^11,12^. It also induced a significant loss in mitochondrial membrane potential and an increase in reactive oxygen species. Synergistic effect of chrysin with cisplatin was observed in HepG2 cells wherein, it elevated the levels of p53, Bax, caspase-3, -8, and -9 as well as reduced the levels of Bcl-2^13^. Thus, inducing intrinsic and extrinsic apoptosis. Maruhashi et al.^14^ reported that chrysin enhances the anticancer potential of drugs in lung squamous cell carcinoma, suggesting its use as an adjuvant in chemotherapy.

Though chrysin is a potent cytotoxic agent, its isolation from plant and honey restricts its upscale production by biological methods for commercial use. Synthesis of chrysin from endophytic fungi can provide an edge to manipulate the physicochemical and biological parameters to increase its yield in an eco-friendly and economical manner. Endophytic fungi have unleashed a new avenue for the search of biologically potent secondary metabolites^15,16^. Recently, Seetharaman et al.^17^ reported chrysin production from an endophytic fungus isolated from passionflower leaves which are already known to produce chrysin.

The marine ecosystem is an extremely diverse reservoir of life harboring myriads of organisms. Extensive drug discovery and secondary metabolite screening investigations over the past few decades have demonstrated marine seaweeds and its associated endophytic fungi to be rich sources of unique bioactive natural products^18^. To the best of our knowledge, there is no report of chrysin production from any marine organisms.

Herein, we report isolation and production of chrysin from a marine endophytic strain, *Chaetomium globosum* isolated from a marine green alga *Chaetomorpha* sp from the intertidal region of the coast of Goa, India.

*Chaetomium* genus is commonly found in terrestrial as well as the marine environment and is widely explored due to its ability to produce an array of bioactive compounds. *C. globosum* isolated from a marine fish was observed to produce novel azophiles and chaetomugilins. The compounds exhibited significant cytotoxicity against leukemia cell lines at IC_50_ values ranging between 30–50 µM. Derivatives of cytochalasin, cytoglobosin and chaetogobosin isolated from marine-algae derived *C. globosum*, displayed cytotoxic activity against A549 cell line^19^. Kumar et al.^20^ reported an apoptotic molecule, flavipin from the endophytic fungus *C. globosum.*

Our study revealed that the total culture ethyl acetate extract of *C. globosum* exhibited potent cytotoxicity on human cancer cell lines^21^. For the first time, the presence of chrysin in the ethyl acetate extracts of a marine fungal endophyte *C. globosum* associated with a green marine alga *Chaetomorpha media* is reported in this study. The mechanism of fungal derived chrysin was also compared with the standard chrysin. This establishes a novel source of this flavone which can be further be explored for yield enhancement by optimization of its production parameters.

## Results

The fungus was associated with the green alga *C. media* and had a lemony yellow color when grown on a PDA plate **(**Fig. 1B). It had velvety texture with filamentous colonies, raised elevation, and undulate margination.

### In vitro cytotoxicity analyses of *C. globosum* ethyl acetate total culture extract by MTT and resazurin assay

As evaluated by MTT assay, *C. globosum* ethyl acetate total culture extract demonstrated concentration-dependent cytotoxicity on A-431, A549, MCF-7, and HeLa cells at 3, 5, 10, 25 µg/mL concentration. It exhibited an IC_50_ value in the range of 4 to 8 µg/mL on all four cell lines (Table 1). The highest cytotoxicity was observed in A549 cells where the IC_50_ value was 4.5 ± 0.1 µg/mL. HeLa, A-431 and MCF-7 cells were also vulnerable to the extract and their IC_50_ values were 8.1 ± 0.1, 4.7 ± 0.1 and 7.9 ± 0.1 µg/mL respectively, indicating significant cytotoxicity. On HEK 293 T cells, the IC_50_ value was > 25 µg/mL. The results indicated the effective cytotoxicity of *C. globosum* ethyl acetate total culture extract on cancer cells. To further confirm the cytotoxicity, resazurin reduction assay was also performed on the same cell lines. The results corroborated well with those obtained from the MTT assay (data not shown).

**Table 1:** The table represents IC_50_ values exhibited by CGEE after 48 h of treatment on various human cell lines as evaluated by MTT assay.

Endophyte	IC_50_ (µg/mL)				
	HeLa	A549	A431	MCF-7	HEK 293 T
*Chaetomium globosum* ethyl acetate extract	8.1 ± 0.1	4.5 ± 0.1	4.7 ± 0.1	7.9 ± 0.1	> 200

The extract demonstrated significant cytotoxicity on human cancer cells while negligible cytotoxic effect on human embryonic kidney cells.

### Chemical composition of *C. globosum* ethyl acetate total culture extract by GC–MS analyses

The total culture ethyl acetate crude extract obtained from the fermentation of *C. globosum* was analyzed by GC–MS. This analysis revealed the presence of myriads of compounds some of which are with known bioactive potential. The chemical components of the derivatized CGEE were studied using GC/MS analysis. Derivatization was performed to increase the degree of volatility of the components for increased fragmentation and better detection. The compounds identified from *C. globosum*, their retention time, and relative abundance (%) are summarized in Table 2. However, many components were unidentified since their spectral data could not be associated with any compound in the NIST database indicating the presence of novel metabolites in the fungal extract. A total of forty-six components were detected in CGEE. Our results indicated that the main constituents of the ethyl acetate total culture extract were 2,2-diethylacetamide (5.1%), hexadecanoic acid (6.1%), 9,12-octadecadienoic acid (Z,Z) (6.7%), trans-9-octadecenoic acid (7.3%), octadecanoic acid (6.5%), chrysin (19.3%) and a propyl ester of octadecanoic acid (4.3%). Azetidine, an alkaloid, was also found in the extract at 2.5%. There seemed to be a predominance of alkanes and fatty acid methyl esters in CGEE even though they were present in low quantities.

**Table 2:** Myco-components detected in ethyl acetate total culture extract of *C. globosum* by GC–MS analysis.

S. no	Name of the component	Chemical formula	Retention time (mins)	Relative abundance (%)
1	Disiloxane, 1,3-bis(chloromethyl)-1,1,3,3-tetramethyl-	C_6_H_16_Cl_2_OSi_2_	7.197	0.9299 ± 0.5
2	Carbamodithioic acid, phenyl-, methyl ester	C_8_H_9_NS_2_	7.291	0.003015 ± 0.1
3	*N*-(Trimethylsilyl)acetamide	CH_3_CONHSi (CH_3_)_3_	7.3048	0.5491 ± 0.8
4	2-Butenoic acid, tert-butyldimethylsilyl ester	C_17_H_36_O_3_Si_2_	7.8719	0.15488 ± 0.1
5	Formamide, *N*,*N*-diethyl-	HCON(C_2_H_5_)_2_	7.886	0.8218 ± 1.1
6	Isoquinolin-6,7-diol, 1-methyl	C_10_H_9_NO_2_	8.2131	1.2960 ± 1.7
7	1-Hexene, 4,5-dimethyl-	C_8_H_16_	8.2312	1.30402 ± 0.1
8	Sulfurous acid, di(cyclohexylmethyl) ester	C_14_H_26_O_3_S	8.2767	0.01376 ± 0.1
9	Azetidine, 1-acetyl-2-methyl-	C_6_H_11_NO	8.2854	2.83459 ± 0.2
10	Disilathiane, hexamethyl	C_6_H_18_SSi_2_	8.348	1.39148 ± 0.1
11	2,4,4-Trimethyl-1-hexene	C_9_H_18_	8.4016	0.4074 ± 0.6
12	Acetamide, *N*-ethyl-	C_4_H_9_NO	8.8449	0.3275 ± 0.5
13	Tris(trimethylsilyl)borate	C_9_H_27_BO_3_Si_3_	9.1013	0.557147 ± 0.1
14	1,2-Bis(trimethylsiloxy)ethane	C_8_H_22_O_2_Si_2_	9.1285	0.8199 ± 1.1
15	Silanol, trimethyl-, carbonate (2:1)	C_7_H_18_O_3_Si_2_	8.7307	0.3330 ± 0.5
16	Acetamide, *N*-ethyl-	C_4_H_9_NO	8.8449	0.3280 ± 0.5
17	1,2-Bis(trimethylsiloxy)ethane	C_8_H_22_O_2_Si_2_	9.1285	0.8200 ± 1.1
18	Acetamide, *N*,*N*-diethyl-	C_6_H_13_NO	9.5024	1.5998 ± 2.2
19	2,2-Diethylacetamide	C_6_H_13_NO	10.0863	2.5606 ± 3.6
20	Tris(trimethylsilyl)carbamate	C_10_H_27_NO_2_Si_3_	10.316	0.1908 ± 0.3
21	*N*,*N*-Diethyl(trimethylsilyl)carbamate	C_8_H_19_NO_2_Si	11.0868	0.2737 ± 0.4
22	Propanoic acid, 2-[(trimethylsilyl)oxy]-, trimethylsilyl ester	C_9_H_22_O_3_Si_2_	11.2173	0.5037 ± 0.7
23	Acetic acid, [(trimethylsilyl)oxy]-, trimethylsilyl ester	C_8_H_20_O_3_Si_2_	11.7152	1.2734 ± 1.8
24	Isophorone	C_9_H_14_O	13.1412	0.3485 ± 0.5
25	Silanol, trimethyl-, phosphate (3:1)	C_9_H_27_O_4_PSi_3_	17.332	0.4860 ± 0.7
26	Glycerol, tris(trimethylsilyl) ether	C_12_H_32_O_3_Si_3_	17.4314	0.8582 ± 1.2
27	Butanedioic acid, bis(trimethylsilyl) ester	C_10_H_22_O_4_Si_2_	18.5644	0.3425 ± 0.5
28	2-(2-Butoxyethoxy)ethoxy-trimethylsilane	C_11_H_26_O_3_Si	19.1932	0.2287 ± 0.3
29	L-Proline, 5-oxo-1-(trimethylsilyl)-, trimethylsilyl ester	C_11_H_23_NO_3_Si_2_	23.7834	0.2626 ± 0.4
30	Tricyclo[5.2.1.0(2,6)]decane, 3-methylene-4-phenyl	C_17_H_20_	27.9234	1.3391 ± 1.9
31	1,4-Benzenedicarboxylic acid, bis(trimethylsilyl) ester	C_14_H_22_O_4_Si_2_	29.9265	0.2885 ± 0.4
32	1-Nonadecene	C_19_H_38_	30.1214	0.1761363 ± 0.2
33	Azelaic acid, bis(trimethylsilyl) ester	C_15_H_32_O_4_Si_2_	30.1519	0.1670219 ± 0.8
34	D-(-)-Ribofuranose, tetrakis(trimethylsilyl) ether (isomer 2)	C_17_H_42_O_5_Si_4_	31.0492	0.6179 ± 0.9
35	Salbutamol, N-trifluoroacetyl-O,O,o-tris(trimethylsilyl)deriv	C_24_H_44_F_3_NO_4_Si_3_	31.309	0.3250 ± 0.5
36	α-D-Allopyranose, pentakis(trimethylsilyl) ether	C_21_H_52_O_6_Si_5_	32.3191	0.2874 ± 0.4
37	Hexadecanoic acid, trimethylsilyl ester	C_19_H_40_O_2_Si	35.1335	3.0713 ± 4.0
38	9,12-Octadecadienoic acid (Z,Z)-, trimethylsilyl ester	C_21_H_40_O_2_Si	38.1093	3.3908 ± 4.7
39	Oleic acid, trimethylsilyl ester	C_21_H_42_O_2_Si	38.2218	1.7593 ± 2.4
40	trans-9-Octadecenoic acid, trimethylsilyl ester	C_21_H_42_O_2_Si	38.2268	7.27069 ± 0.8
41	Octadecanoic acid, trimethylsilyl ester	C_21_H_44_O_2_Si	38.7029	3.2747 ± 4.6
42	Stearic acid, 2-(1-octadecenyloxy)ethyl ester, (Z)-	C_38_H_74_O_3_	42.4672	1.2604 ± 1.8
43	Hexadecanoic acid, 2,3-bis[(trimethylsilyl)oxy]propyl ester	C_25_H_54_O_4_Si_2_	44.2692	0.4854 ± 0.7
**44**	**Chrysin, bis(trimethylsilyl) ether**	**C**_**21**_**H**_**26**_**O**_**4**_**Si**_**2**_	**44.6518**	**12.8317 ± 3.5**
45	Octadecanoic acid, 2,3-bis[(trimethylsilyl)oxy] propyl ester	C_27_H_58_O_4_Si_2_	47.0836	2.1723 ± 3.0
46	.psi.,.psi.-Carotene, 1,1′,2,2′-tetrahydro-1,1′-dimethoxy-	C_42_H_64_O_2_	52.9052	0.0880 ± 0.1

Chrysin (highlighted in bold) was one of the myco-components detected in the ethyl acetate total culture extract of *C. globosum*. The components were identified by comparing the spectrum of the unknown components with the spectrum of reported components in the National Institute Standard and Technology (NIST) library. The relative abundance of each component was calculated by comparing its average peak area to the total area contributed by all the components.

### Effect of fermentation and extraction parameters on biomass and production of cytotoxic metabolites

#### Growth curve of *C. globosum* and cytotoxic metabolites production in suspension culture of *C. globosum*

**Figure 2:**
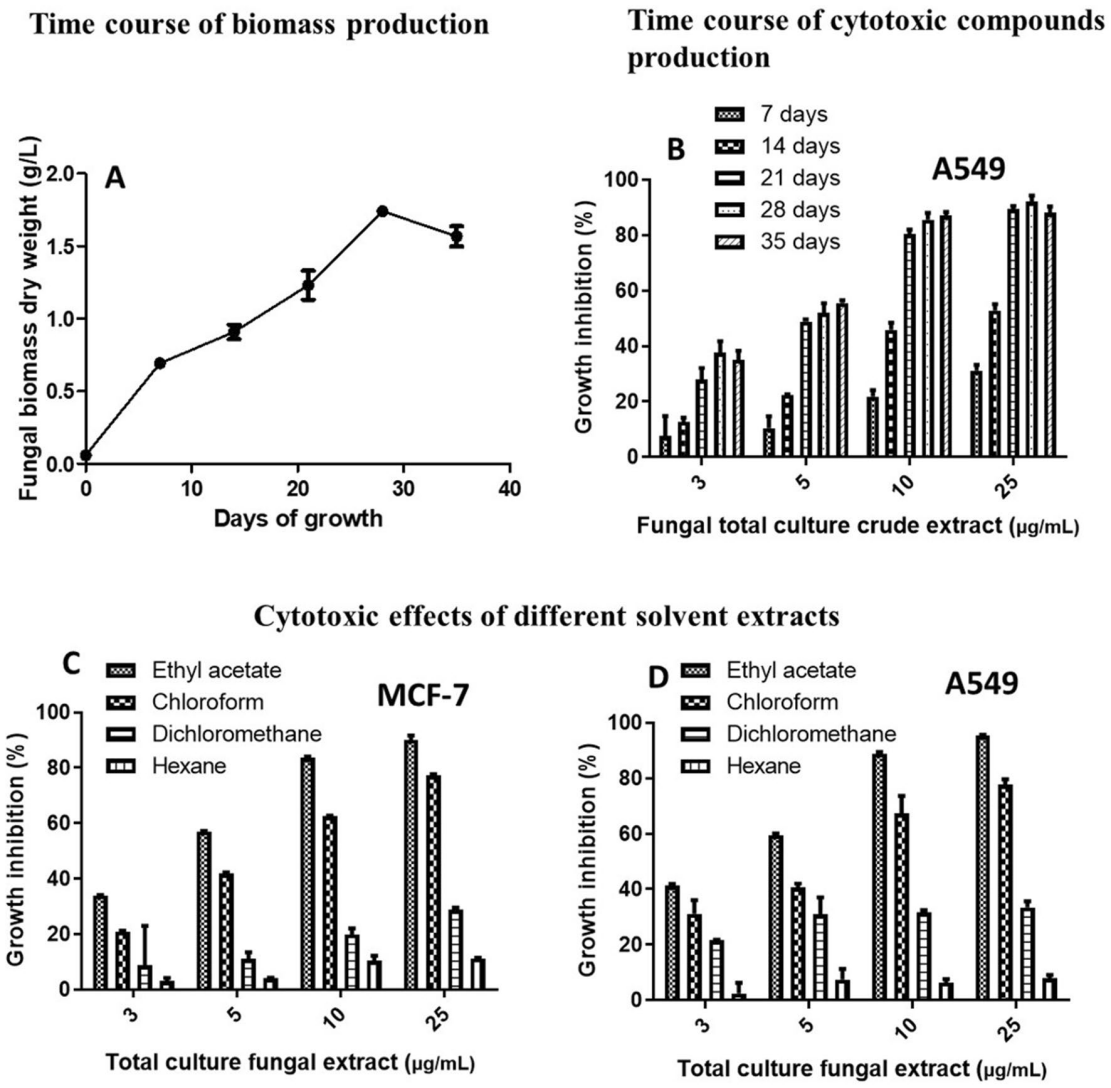
Optimization of parameters for the extraction of cytotoxic secondary metabolites. (**A**) Growth curve analyses of *C. globosum* grown in potato dextrose broth for thirty-five days. Mycelial dry weight was calculated every seven days. (**B**) Evaluation of optimum incubation time interval for extraction of cytotoxic secondary metabolites from *C. globosum*, evaluated by MTT assay on A549 cells. Optimization of appropriate organic solvent for total extraction of cytotoxic metabolites from *C. globosum* by MTT assay on (**C**) MCF-7 and (**D**) A549 cells.

The incubation time interval is a crucial variable for the extraction of cytotoxic compounds. Hence, *C. globosum* growth curve was plotted by incubating it for a period of 7, 14, 21, 28, and 35 days. As depicted in Fig. 2A, an exponential growth pattern was observed until 28 days after which the growth reached a stationary phase. Maximum biomass production of 1.8 ± 0.3 g/L was reached during the 28th day of incubation after which a declining phase was observed. Maximum cytotoxicity was also demonstrated by the same culture ethyl acetate extract on MCF-7 and A549 cells at 3–25 µg/mL as observed by the MTT assay (Fig. 2B). These results implicated maximum cytotoxic metabolite production in *C. globosum* grown for 28 days in 200 mL of PDB. Our observation corroborates with the fact that the synthesis of secondary metabolites usually occurs in the stationary phase.

### Effect of solvent on the extraction of cytotoxic secondary metabolites

Solvent extraction is an imperative step in recovering and isolating secondary metabolites from the fungal material. The polarity of the solvent gives an idea about the chemical nature of the components in that extract. Hence, the solvent extraction of *C. globosum* was performed after 28 days of inoculation in PDB as optimized earlier. The solvents used were hexane, dichloromethane, chloroform, and ethyl acetate. Maximum extraction of cytotoxic compounds was observed in the ethyl acetate extract as exhibited by the highest percentage of cell death after 48 h in MCF-7 and A549 cells by MTT assay at concentrations ranging from 3 to 25 µg/mL. Hexane extract exhibited the least cytotoxicity of 20% in the same cells (Fig. 2C,D).

Experimental observations of these optimization studies revealed that 28 days old culture of *C. globosum*, extracted in ethyl acetate demonstrates the highest cytotoxicity in MCF-7 and A549 cells. Hence these parameters were fixed to prepare *Chaetomium globosum* ethyl acetate extract (CGEE).

### Mechanism of CGEE cytotoxicity by flow cytometry

**Figure 3:**
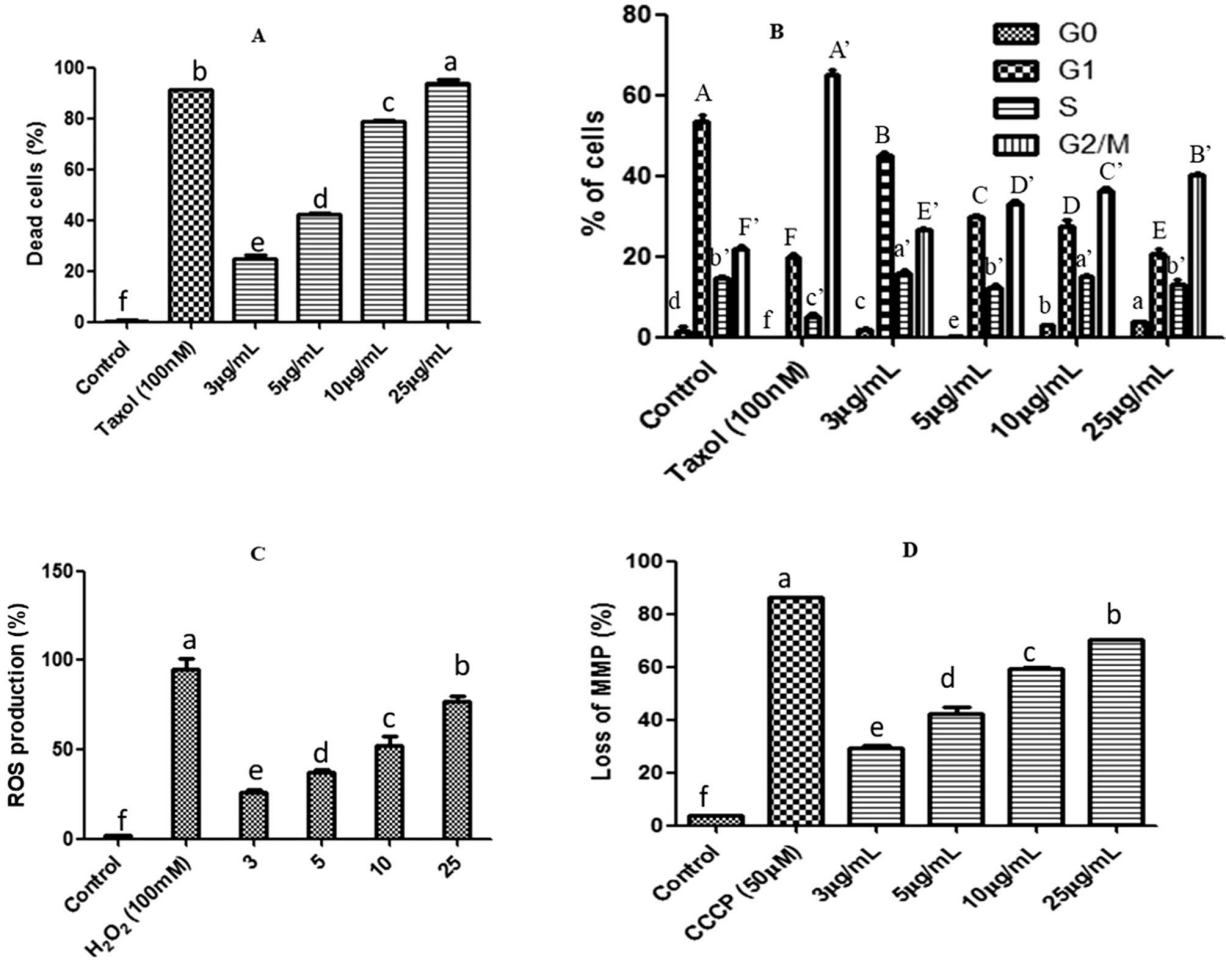
Cytotoxic effect of CGEE on MCF-7 cells: (**A**) MCF-7 cells treated with a range of concentrations of CGEE for 24 h. The percentage of live and dead cells was evaluated by propidium iodide (PI) staining and quantified by FACS. 100 nm Paclitaxel treated cells served as positive control. (**B**) Effect of CGEE treatment on cell cycle phase distribution of MCF-7 cells after 24 h. The percentage of cells in each phase was evaluated by PI staining and quantified by FACS. CGEE induced G2/M phase arrest. (**C**) Evaluation of ROS level in response to 24 h of CGEE treatment in MCF-7 cells determined by DCFH-DA staining followed by FACS analysis. (**D**) Effect of 24 h of CGEE treatment on MMP dynamics in MCF-7 cells measured by JC-1 staining followed by FACS analysis. Flow cytometry data were quantified using the CytExpert 2.0 software. Means sharing different letters differ significantly from each other at p ≤ 0.05.

CGEE induced cytotoxicity was studied by FACS analyses (S1). Live and dead cell populations were quantitatively detected and measured by the PI fluorescence using FACS. The cytotoxic activity of CGEE was examined on MCF-7 cells at 3–25 µg/mL concentrations. There was a concentration-dependent increase in the percentage of dead cells corroborating with MTT results. The highest percentage of dead cells was observed at the 25 µg/mL of CGEE which was comparable with the positive control (Fig. 3A).

Cell cycle progression of MCF-7 cells was analyzed by PI staining after treatment with 3–25 µg/mL of CGEE (Fig. 3B). On treatment with CGEE, the population of cells in the G2M phase significantly increased from 21.9 ± 0.1% in untreated to 41.3 ± 2.1% after treatment of 25 µg/mL indicating a G2M phase arrest. An increase in the sub-G1 population at 10 and 25 µg/mL of CGEE corroborate with the results of PI live dead assay indicating an increase in the percentage of dead cells.

The generation of reactive oxygen species (ROS) was studied by DCFH-DA staining. Treatment with four concentrations of CGEE induced a steady increase in ROS production up to 71.1 ± 1.3% in MCF-7 cells after 24 h of treatment (Fig. 3C). Since the generation of reactive oxygen species (ROS) is linked with MMP loss, CGEE could also induce loss in MMP was studied after 24 h of treatment on MCF-7 cells. The percentage of MCF-7 cells with depolarized mitochondria increased from 28.6 ± 1.6% at 3 µg/mL to 71.5 ± 1.0% at 25 µg/mL (Fig. 3D).

### Isolation, structure elucidation and anti-cancer activity of Chrysin purified from *Chaetomium globosum*

Chrysin was detected as one of the volatile compounds in the CGEE by GC–MS analysis with 99% probability*.* Hence, this compound was studied by subjecting CGEE to thin-layer chromatography. Chrysin procured from Sigma, India (SChr) was used as a standard.

### Single-step purification of chrysin by TLC

**Figure 4:**
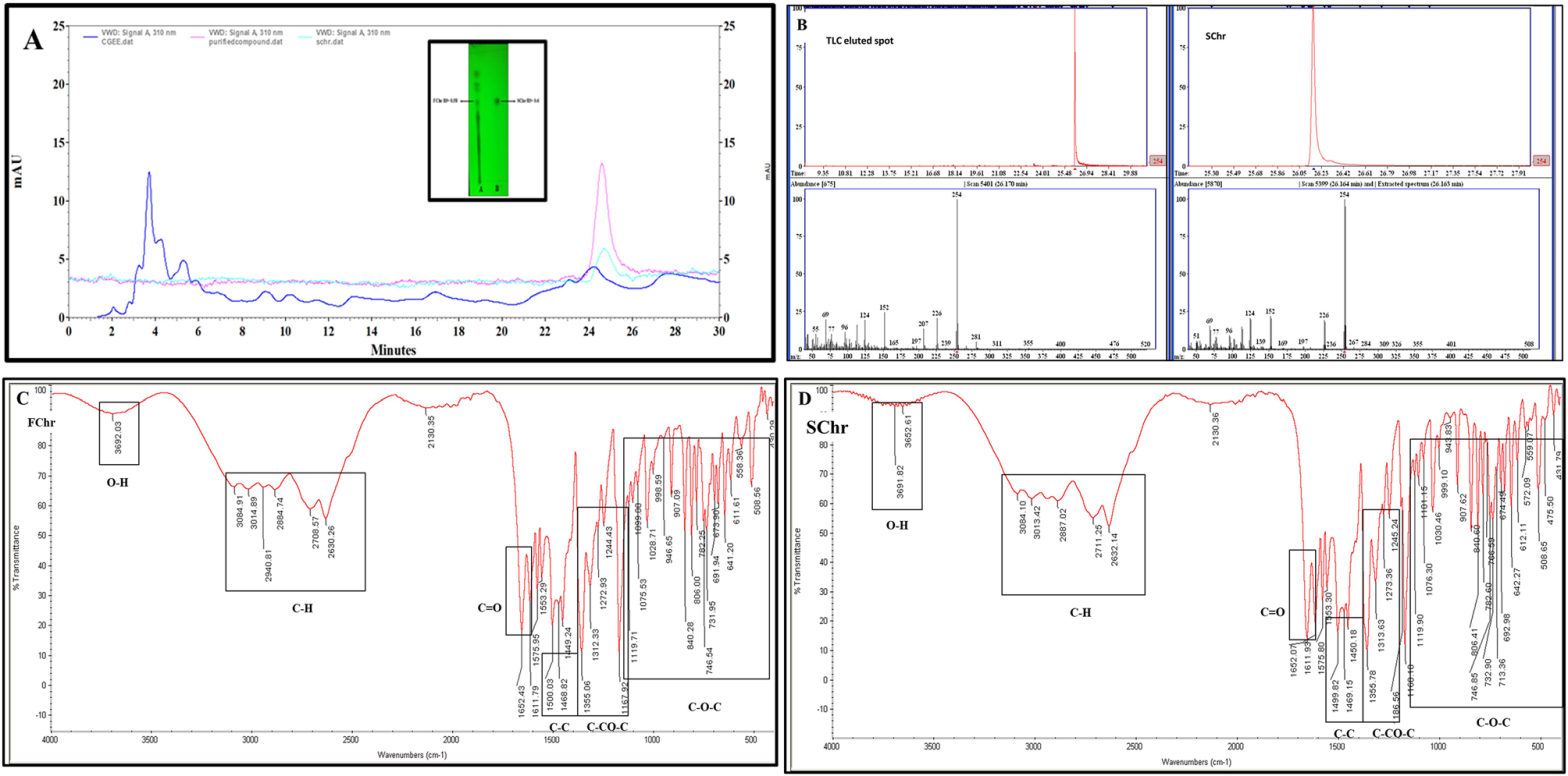
Purification and structure elucidation of compound Chrysin from CGEE. (**A**) Inset: TLC analysis of CGEE and standard Chrysin (SChr). Overlaid HPLC chromatograms of TLC spot (purified compound) at retention time 24.600 min, SChr showing a peak at retention time 24.687 min and CGEE showing a peak at retention time 24.387 min. The analysis was conducted on an Agilent C18 column using a mobile phase of 50% methanol (**A**) and 50% acetonitrile/water (**B**) at a flow rate of 1.0 mL/min and absorbance was monitored at 310 nm wavelength. (**B**) Comparison of GC–MS analyses of TLC eluted spot and SChr. Single peaks were obtained for both the samples at retention time 26.170 for TLC eluted spot and 26.164 for SChr. Comparison of the FT-IR spectrum of (**C**) anticipated fungal chrysin (FChr) and (**D**) SChr indicating the presence of similar functional groups in both the compounds.

Out of the nine solvent systems used to optimize the separation and purification of chrysin, solvent system number 3 (toluene: ethyl acetate: acetic acid 36:12:5) gave the best resolution when viewed under 254 nm and hence was selected for further purification. The R_f_ value of SChr was 0.6 while a corresponding spot in CGEE had a R_f_ value of 0.58 indicating the separation and purification of chrysin from *C. globosum* (Fig. 4A inset). Further, preparative TLC was performed to obtain more quantity of the compound.

### GC–MS analyses of TLC eluted spot

The preparative TLC spot indicating separated chrysin was scrapped and dissolved in methanol. A single peak at RT 26.170 min was found for the TLC spot while SChr exhibited a peak at RT 26.164 min (Fig. 4B). The NIST database matched this chromatogram with that of Chrysin with 98% probability.

### Structural characterization of purified chrysin by UV–Visible spectroscopy, HPLC, LC–MS/MS, NMR and IR spectroscopy

The purified compound was a lemon yellow colored amorphous powder. UV–Vis spectroscopy revealed that the λ_max_ of the compound was at 310 nm. The λ_max_ of SChr was also observed at 310 nm (S2). HPLC analysis of the purified compound revealed a single peak at a retention time of 24.60 min while the SChr peak was detected at 24.69 min. Among the many peaks detected in the HPLC chromatogram of CGEE, a peak was detected at 24.39 min. An overlaid chromatogram of the three samples indicates the presence of chrysin in CGEE (Fig. 4A).

Infrared spectroscopy (IR) was used to identify the functional groups present in the purified compound and compared with the SChr. As evident from the spectrum (Fig. 4C,D) a vibrational stretch corresponding to the O–H group was observed at 3,692.03 cm^−1^ for purified chrysin and 3,691.82 cm^−1^, 3,652.61 cm^−1^ for SChr. The C–H stretching was noted in the range between 2,630.26 cm^−1^ to 3,084.91 cm^−1^ in purified chrysin, while this was observed between 2,632.14 cm^−1^ to 3,084.10 cm^−1^ in SChr. A strong vibrational stretch of C=O was observed at 1652.43 cm^−1^ and 1611.79 cm^−1^ in purified chrysin and 1652.07 cm^−1^ and 1611.93 cm^−1^ in SChr. The C–C bending was observed in the range of 1,500.03 cm^−1^–1,468.82 in purified chrysin and SChr. Vibrational stretching corresponding to C–CO–C stretching was detected at 1,355.06 cm^−1^–1,167.92 cm^−1^ in purified chrysin and SChr. Finally, C–O–C aromatic bending vibrations were noted between 1,119.71 cm^−1^ to 475.50 cm^−1^ in purified chrysin and SChr. The FTIR spectrum of purified chrysin matched significantly with that of the SChr. The data obtained was in coherence with earlier reported IR spectra of free chrysin as reported by Faize et al.^22^, Sathishkumar et al.^23^, Seetharaman et al.^17^, and Sulaiman et al.^24^.

**Figure 5:**
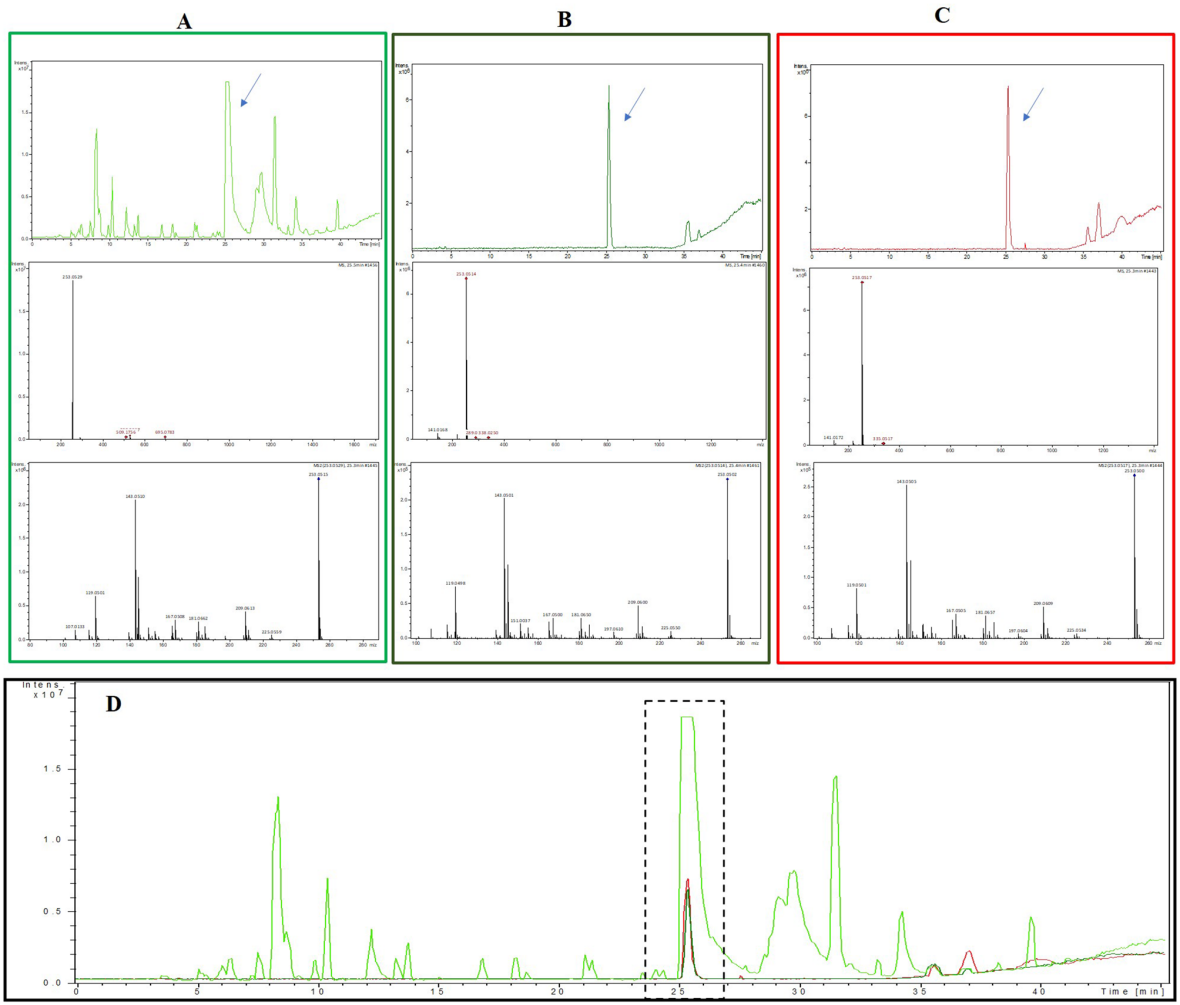
LC–MS/MS analysis of CGEE (**A**): an intense peak at 25.5 min, MS and MS/MS analysis of that peak demonstrated a precursor ion at m*/z* 253.0529 [M−H]. FChr (**B**): single peak obtained at 25.4 min, MS and MS/MS analysis demonstrated precursor ion at *m/z* 253.0514 [M-H]. SChr (**C**): single peak obtained at 25.3 min, MS and MS/MS analysis demonstrated a precursor ion at 253.0517 [M−H] (**D**). Overlaid chromatograms of CGEE, FChr and SChr where dotted box indicates presence of chrysin at retention time ~ 25.5 min.

On LC–MS/MS spectrum of CGEE performed in negative ionization mode, a peak at RT 25.5 min demonstrated a precursor ion at m*/z* 253.0529 [M−H] (Fig. 5A). As presented in Fig. 5B,C, peaks showing precursor ion at *m/z* 253.0514 [M−H] and 253.0517 [M−H] were observed for FChr (RT-25.4 min) and Schr (RT-25.3 min), respectively. The molecular weight of chrysin is 254.0579 (C_15_H_10_O_4_). Apart from a similar retention time (Fig. 5D), MS/MS data revealed the same fragmentation pattern for all the three peaks. Further, mass-based search followed by spectral matching of MS/MS fragments using MetFrag and Metlin databases were used to putatively identify the compound at peak ~ 25.5 min as chrysin. Table 3 shows the MS/MS fragments of precursor ions and their spectral matching scores with chrysin.

**Table 3:** MS/MS fragments of precursor ions and their spectral matching scores with chrysin.

Samples	Retention time (min)	Precursor [M−H]	MS/MS fragments	MetFrag Score	Metlin Score	Precursor ΔPPM
CGEE	25.5	253.0529	119, 143, 167, 181, 209, 225, 253	1	100	9.08
FChr	25.4	253.0514	119,143,167,181,197,209, 225, 253	1	100	3.00
SChr	25.3	253.0517	119,143,167,181,197,209, 225,253	1	100	4.38

**Figure 6:**
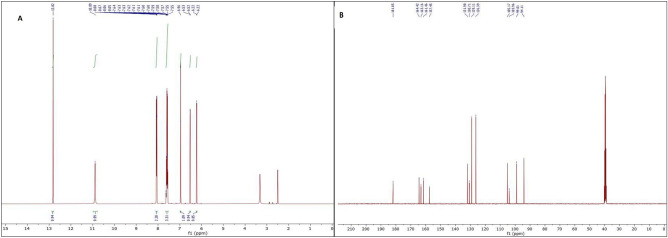
The ^1^H NMR (**A**) and ^13^C NMR (**B**) spectra of fungal chrysin produced by the endophyte *Chaetomium globosum.*

The identity of the purified compound as fungal chrysin was confirmed by NMR analysis. ^1^H NMR and ^13^C NMR spectra of FChr are given as Fig. 6.

FCHR:^1^H NMR (400 MHz, DMSO-*d*6) δ (ppm), 12.82 (1H, s, OH-5), 10.89 (1H, s, OH-7), 8.06 (2H, dd, *J* = 8.1 Hz and 1.6 Hz, H-2′, 6′), 7.58 (3H, m, H-3′, 4′, 5′), 6.96 (1H, s, H-3), 6.52 (1H, d, *J* = 2.1 Hz, H-8), 6.22 (1H, d, *J* = 2.0 Hz, H-6).

^13^C NMR (101 MHz, DMSO-*d*6) δ (ppm),163.16 (C-2), 105.17 (C-3), 181.85 (C-4), 161.46 (C-5), 99.01 (C-6), 164.42 (C-7), 94.11 (C-8), 157.45(C-9), 103.96 (C-10), 130.71(C-1′), 126.39 (C-2′, 6′), 129.11 (C-3′, 5′), 131.98(C-4′).

^1^H and ^13^C chemical shifts of FChr were same as that of SChr (Supplementary data file 1 and Supplementary data file 2), which further confirmed that the fungal metabolite was chrysin.

### Cytotoxic effect of FChr and SChr on MCF-7 and HEK 293 T cells by MTT assay

**Figure 7:**
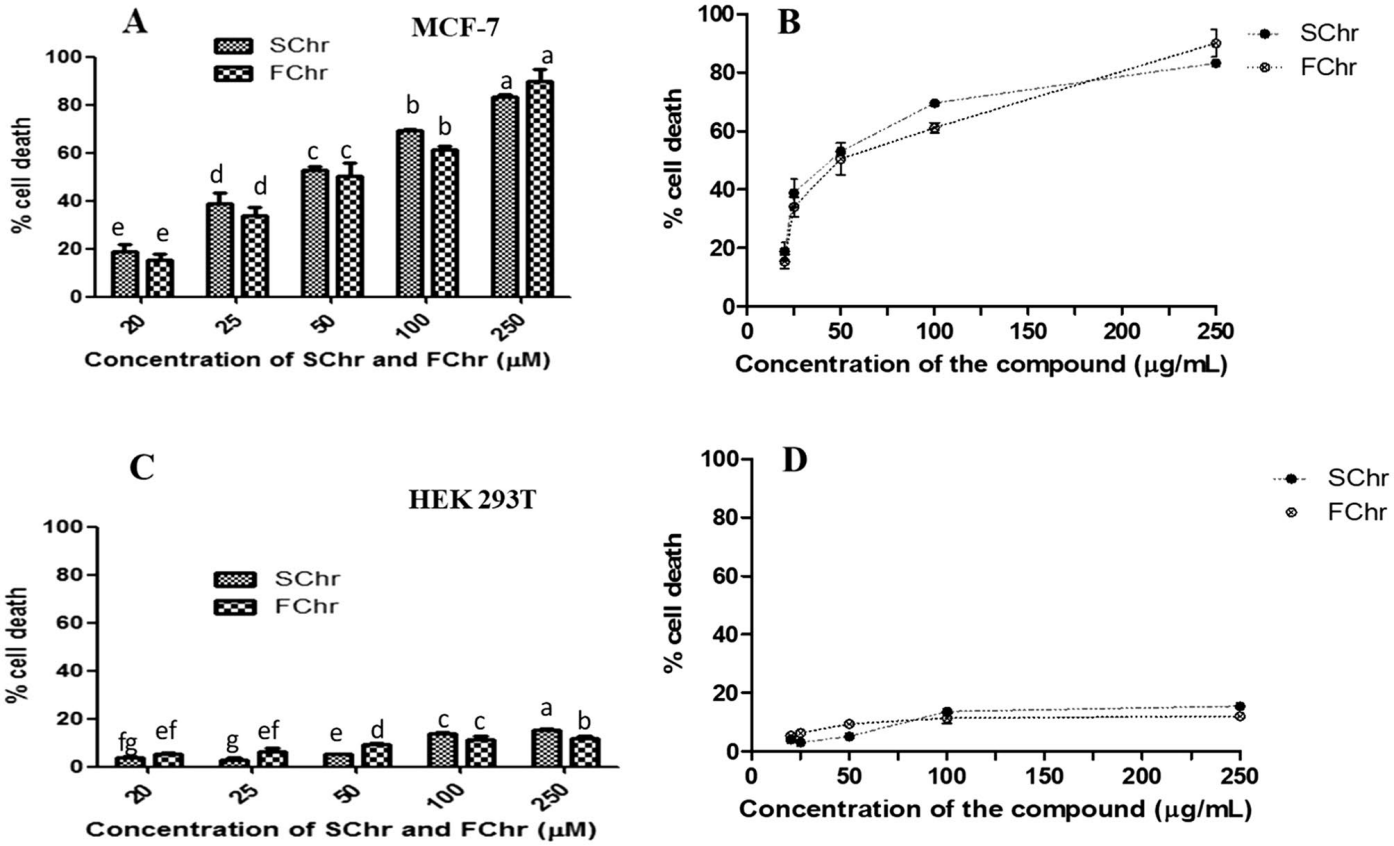
Evaluation of the cytotoxic activity of purified fungal chrysin (FChr) and standard chrysin (SChr) after 72 h of treatment at a range of concentrations ranging from 20 to 250 µM by MTT assay on (**A**) MCF-7 and (**C**) HEK 293 T cells. Dose response curve of FChr and SChr induced cytotoxicity in (**B**) MCF-7 cells and (**D**) HEK 293 T cells. Means sharing different letters differ significantly from each other at p ≤ 0.05.

The in vitro cytotoxic potential of FChr and SChr was evaluated against MCF-7 and HEK 293 T cell lines by MTT assay after 72 h of exposure (24 and 48 h MTT data not shown). The cytotoxicity exhibited by the two compounds was concentration-dependent after 72 h of treatment. Moderate cytotoxicity was observed after 24 and 48 h of treatment. MCF-7 was the only human cancer cell line chosen since it is known to be most vulnerable to chrysin after 72 h. SChr exhibited an IC_50_ value of 48.5 ± 1.6 µM while that of FChr was 49.1 ± 0.6 µM (Fig. 7A,B). Negligible cytotoxicity was observed in HEK 293 T cells hence its IC_50_ value will be > 250 µM. (Fig. 7C,D). The selectivity index demonstrated by FChr and SChr was 5.10 and 5.15 respectively.

### Antiproliferation and apoptosis induction by purified chrysin.

**Figure 8:**
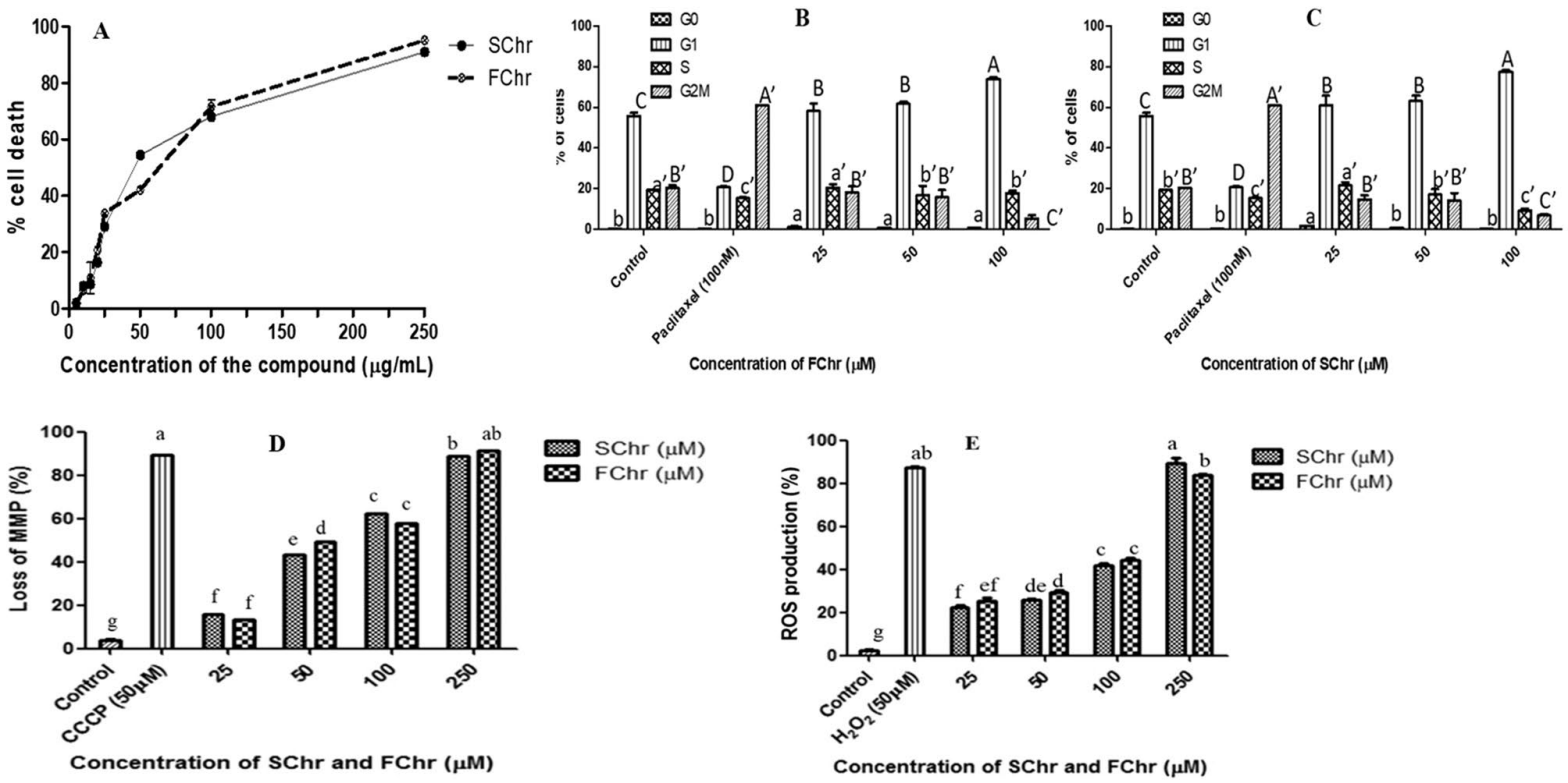
Validation of cytotoxic effects of purified FChr and SChr on MCF-7 cells: (**A**) The curves indicate the percentage of cell death in response to a range of concentrations of FChr and SChr after 72 h of treatment by PI staining followed by FACS analysis. (**B**) The bar diagram indicates the distribution of cells in the phases of the cell cycle in response to 72 h of FChr and (**C**) SChr treatment at a range of concentrations. Both the treatments induced G1 phase arrest in MCF-7 cells. (**D**) Effect of FChr and SChr on MMP dynamics in MCF-7 cells after 72 h. (**E**) Change in ROS levels in MCF-7 cells in response to 24 h of treatment of FChr and SChr. Flow cytometry data were quantified using the CytExpert 2.0 software. Means sharing different letters differ significantly from each other at p ≤ 0.05.

**Figure 9:**
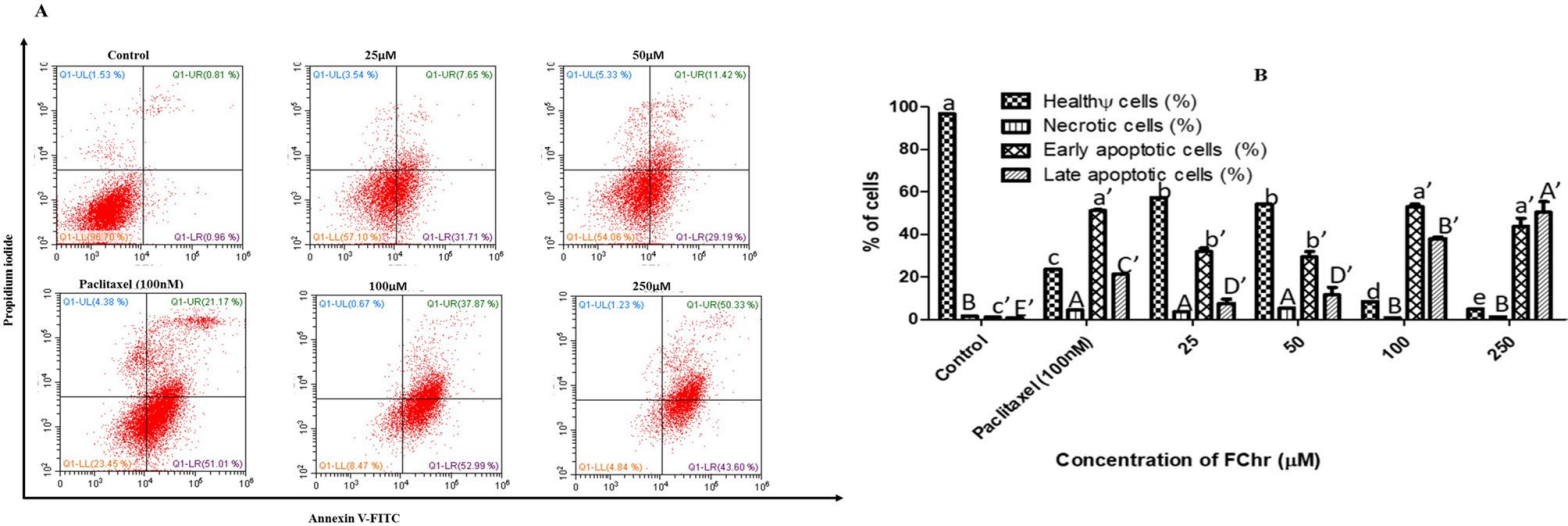
FACS analysis of MCF-7 cells stained with Annexin-V FITC/PI after treatment with different doses of FChr for 72 h. Flow cytometry data were quantified using the CytExpert 2.0 software. Means sharing different letters differ significantly from each other at p ≤ 0.05.

Biological validation of purified chrysin from CGEE was done on MCF-7 cells by FACS analyses (Figs. 8, 9, S3).

### PI live dead analyses

Dose–response of FChr and SChr on MCF-7 cells was plotted by PI live dead assay after 72 h of treatment. Cells were treated with eight concentrations ranging from 5 to 250 µM of FChr and SChr. The FACS analysis showed a concentration-dependent effect of both FChr and SChr on the viability of MCF-7 cells (Fig. 8A). The percentage of dead cells increased up to 80.6 ± 1.6% and 93.4 ± 2.8% for SChr and FChr at 250 µM respectively. Paclitaxel (100 nM) was used as a positive control which exhibited 90.9 ± 2.0% death.

### Cell cycle phase distribution

MCF-7 cells treated with three concentrations (25, 50, 100 µM) of FChr and SChr for 72 h were analyzed for cell cycle phase distribution by PI staining (Fig. 8B,C). Both the compounds arrested the population of cancer cells in the G1 phase. The untreated control displayed 55.2 ± 0.5% in the G1 phase, while, with an increase in the concentration of SChr and FChr, the G1 phase population increased to 74.0 ± 0.1% and 78.2 ± 1.7% respectively. Paclitaxel (100 nM) served as positive control arresting the cells very prominently in the G2M phase.

### Effect of purified chrysin on mitochondrial functions of MCF-7 cells

In an attempt to assess whether the inhibitory effects on cell proliferation are associated with a change in MMP loss and ROS production, FChr and SChr treated cells were subjected to JC-1 and DCFH-DA staining. As depicted in Fig. 8D, MMP loss was measured by reduction in red to green fluorescence ratio after probing with cationic dye JC-1. Loss of MMP in MCF-7 cells treated with FChr and SChr was observed after 72 h of treatment. A concentration-dependent increase in depolarization of mitochondria was observed. At the highest concentration of SChr (250 µM) and FChr (250 µM), 87.4 ± 2.0% and 91.6 ± 0.8% loss of MMP was observed. At the IC_50_ value, a ~ 50% loss in MMP was observed. CCCP treated cells served as a positive control. In response to a 72 h long treatment of FChr and SChr in MCF-7 cells, ROS production was detected in a concentration-dependent manner (Fig. 8E). At 50 µM concentration in both the treatments, the ROS generation was less than 30%. However, at higher concentration of FChr (250 µM) demonstrated 82.5 ± 1.8% while SChr exhibited 90.3 ± 2.5% ROS production respectively. CCCP (50 µM) treated cells exhibited ~ 80% MMP loss while H_2_O_2_ (100 mM) treated cells exhibited ~ 90% ROS production and served as positive controls for both the experiments.

### Evaluation of apoptosis induction by FChr in MCF-7 cells by AnnexinV FITC/PI staining assay

Apoptosis induction by FChr was further confirmed by FACS analysis of annexin V-FITC/PI double staining. To distinguish between cells undergoing apoptosis and necrosis, the cells were counterstained with PI, as annexin V can bind to necrotic cells too. The lower left quadrant represents the double negative indices since living, healthy cancer cells are impermeable to PI, with the borderline presence of PS on its extracellular surface. However, the lower right quadrant represents the early apoptotic cells that are just positive for annexin V-FITC due to the flipped-out PS and an unbroken cell membrane. The upper right quadrant represents cells in the late stages of apoptosis owing to loss of membrane integrity and hence are double-positive indices for PI and annexin V. Finally, the upper left quadrant includes the necrotic cells which are typically positive only for PI, owing to membrane disintegration and cellular content leakage, resulting in staining of only the fragmented DNA^25^. As indicated by FACS analysis (Fig. 9), MCF-7 cells treated with four concentrations (25, 50, 100, 250 µM) of FChr for 72 h, showed considerable apoptosis. At 100 µM dose, maximum cells (53.8 ± 2.0%) showed early apoptosis as indicated by the scatter plot clustering in the lower right quadrant. At the highest concentration of FChr ((250 µM), only 4.9 ± 0.9% cells remained healthy and live while about 50% entered the late apoptotic stage.

## Discussion

The death toll of cancer is increasing exponentially. By 2050, it is expected to rise to 17.5 million because of the increasing exposure to carcinogenic agents, inadequate and expensive cancer chemotherapies as well as their harmful side effects. Hence, natural product research opens a wide arena for the discovery of new economical chemotherapeutic compounds from various sources^26^. Marine algae associated endophytic fungi community is looked upon as a hopeful source of potent anti-cancer compounds and as an alternative source of plant-based products. The Konkan coast of India is a relatively unexplored environment for endophytic fungi and their bioactive compounds^18^. Earlier studies have acknowledged marine algicolous endophytic fungi as hot factories of bioactive compounds because of their unique environmental settings^27^. This study has been performed to evaluate the cytotoxic potential of a marine-derived endophytic fungus and to explore its unique productome which can either be novel or have the capacity to replace the existing sources of the natural product.

In this study, endophytic fungus *C. globosum* was isolated from a marine green alga^18^. A total of 26 different fungi were isolated and screened for their cytotoxic potential in our earlier study^18^. *C. globosum* isolated from alga *Chaetomorpha* sp. showed significant cytotoxicity against cancer cell lines. The phylogenetic tree constructed showed its closeness to other *Chaetomium* sp. including *Chaetomium subaffini*, *Chaetomium cochlioides*, *Chaetomium pseudocochlioides,* and *Chaetomium acropullum*^18^. The genus *Chaetomium* has been a prolific source of a wide array of chaetoglobosins, epipolythiodioxopiperazines, azaphilones, xanthones, anthraquinones, chromones, depsidones, steroids, and terpenoids. These compounds are known for their anti-cancer, anti-malarial, and antibiotic activities^28^. An anti-cancer compound Chaetopyranin was extracted by Wang et al.^29^, from *C. globosum* associated with a marine red alga. The endophytic fungus, *C. globosum* was earlier isolated from a marine green alga *Chaetomorpha* sp sampled from the Palolem coast, Goa-India. The fungus had a characteristic lemon-yellow color when grown on PDA plate indicating the production of flavonoids or terpenoids^30^. Further, solvent and fungal growth time were also optimized to get the maximum yield of bioactive agents in the crude extract. Organic solvent systems and incubation time for the fungus are important parameters to optimize and enhance the yield of bioactive substances^31^. Organic solvents can provide an idea about the polarity of the active ingredients, whereas incubation time optimization can help in enhancing the production of required secondary metabolites^32^. The highest cytotoxic activity was obtained in the extract of *C. globosum* grown for 28 days. The extract exhibited an IC_50_ value of 7.9 ± 0.1 µg/mL. The results indicated the presence of potent cytotoxic compounds in the extract. The cytotoxicity assays confirmed that CGEE can inhibit the proliferation and survival of all four cancer cell lines tested as indicated by its very low IC_50_ values. The difference in IC_50_ values on all four cell lines was not very significant thus illustrating the potential of CGEE cytotoxicity on a wide range of cancer cell lines.

In this study, maximum cytotoxicity was observed in the ethyl acetate crude extract of *C. globosum* grown for a period of 28 days corroborating with the results obtained earlier in other studies^33^. This indicated that the extract could contain compounds of semi-polar nature with interesting anticancer activity. The CGEE extract showed better cytotoxic activity against multiple cancer cell lines than many crude extracts earlier reported in literature^34–36^. There have been multiple types of secondary metabolites studied and characterized by the endophytic fungus *C. globosum*. Chaetophyranin 1, isolated from *C. globosum* associated with a red alga, exhibited an IC_50_ value of 39.1 µg/mL on A549 cells among others^34^. Cytochalasin based compounds have also been reported from this fungus associated with a grass *Imperata cylindrica*^37^*.* Cytogoblosins, the very popular fungal alkaloids were reported from *C. globosum* associated with a marine green alga *Ulva pertusa.* These alkaloids exhibited prominent cytotoxicity with IC_50_ values of 2.55 µM against the A549 cells^38^. Such an array of potent compounds certainly justifies the cytotoxicity of CGEE with very low IC_50_ values against all the four cell lines. GCMS is one of the most common and inexpensive spectroscopic techniques routinely used for the identification of chemical constituents of an extract. GCMS study of CGEE revealed several bioactive compounds present in the crude extract including octadecanoic acid, chrysin, and azetidine. A study had reported that octadecanoic acid derivatives are important elements in modulating the production of antibiotic compounds and thus will always be an integral part of secondary metabolite profile^39^. As *C. globosum* ethyl acetate extract illustrates to be a cocktail of a variety of these methyl esters along with a dihydroxyflavone, presumably they should be responsible for its prominently low IC_50_ values on the four cancer cell lines.

The underlying mechanism of cytotoxicity against MCF-7 cells was also elucidated by measuring ROS and MMP production in cancer cells treated with extract and chrysin. CGEE induced concentration-dependent apoptotic cell death by destabilizing MMP in a ROS dependent manner^40,41^.

Further search for cytotoxic secondary metabolites from this fungus may lead to the isolation of novel and very potent cytotoxic compounds.

Chrysin detected in the GC–MS and LC–MS/MS analyses of CGEE was purified by TLC and further characterized by chromatographic and analytical methods such as UV–vis spectroscopy, FTIR spectroscopy, LC–MS/MS, NMR, GC–MS, and HPLC. The biological activity of fungal chrysin was tested in vitro using various cytotoxicity assays against MCF-7 breast cancer cells in comparison with the standard chrysin, resulting in comparable activities. The SI demonstrates the differential action of a compound. An SI value < 2 indicates general toxicity, while > 2 indicates better selectivity^42^. Interestingly, it was observed in this study that the SI of both FChr and SChr was ~ 5 indicating a high degree of cytotoxic selectivity. As reported in the literature, fungal chrysin showed interesting cytotoxic potential by inducing apoptosis as observed by annexin/FITC staining. Earlier studies have suggested that the anti-proliferative effects of honey, propolis, and passionflower extracts are mainly due to chrysin. Seetharaman et al.^17^ demonstrated the similar cytotoxic activity of chrysin isolated from endophytic fungi in comparison to standard chrysin against MCF-7 cells. Rasouli and Zarghami^43^ found in their study that chrysin had an IC_50_ value of 44.78 µM against T47D breast cancer cells after 48 h of incubation. In South Korea, China, and Japan, chrysin isolated from *Scutellariae Radix* has been used to treat pneumonia, jaundice, and breast mass. The IC_50_ dose of chrysin on prostate carcinoma cells was determined to be 24.5 ± 0.1 µM after 48 h of treatment. The neoplastic potential of chrysin is very well known^8–14^.

Herein we report, for the first time the production of chrysin by an endophytic fungus *C. globosum*, isolated from marine macroalga. The cytotoxic potential of fungal chrysin was significantly comparable to that of the standard chrysin. Though chrysin has earlier been reported in honey and propolis tree, the upscale production of chrysin from plant and related agents is very difficult owing to their slow growth rate and difficulty in optimizing growth parameters. Synthesis of chrysin from a marine-derived endophytic fungus opens a new field to enhance its production by simpler and cost-effective methods of growth optimization such as stress induction, media optimization, and treatment with elicitors. This study also indicates the presence of a vast reservoir of terrestrial natural products in the marine ecosystem. Endophytic fungi associated with marine algae represent a relatively unexplored reservoir of bioactive diversity. These organisms are promising alternative sources of plant-derived compounds and growth parameters can be manipulated by various means to generate myriads of bioactive metabolites, to amplify the productivity of a compound or to produce completely novel bioactive compounds^44,45^. Physicochemical and media optimization of *C. globosum* is underway to enhance the production of chrysin in an economical and eco-friendly manner. Bioavailability of chrysin is a problem that needs to be resolved to proceed further with this compound to use it as commercially as an anticancer compound.

## Conclusion

In this study, the crude extract of a marine-derived fungus *C. globosum* exhibited significant cytotoxicity against cancer cell lines. It was also evidenced for the first time that a marine alga associated fungal endophyte, *C. globosum* produced chrysin, a dihydroxyflavone. Fungal chrysin demonstrated an apoptotic cell death in cancer cell line MCF-7 in a ROS dependent manner. Thus, it can be concluded that the bioactive secondary metabolite productome of marine algae associated fungal endophytes is extremely extensive and can be exploited in drug discovery of anti-cancer compounds. It also presents immense scope for the enhanced production of potential anti-cancer compounds.

## Materials and methods

### Chemicals

Fungal growth media, Potato Dextrose Agar and Potato Dextrose Broth (PDA and PDB), and trypsin–EDTA were purchased from Himedia, India. Antibiotics and 3-(4, 5-dimethylthiazole-2yl)-2, 5-diphenyl tetrazolium bromide (MTT) reagent were purchased from SRL-Ranbaxy. Ethyl acetate, hexane, chloroform, dimethyl sulphoxide (DMSO), deuterated DMSO and dichloromethane were procured from Merck, India. Dulbecco’s Modified Eagle’s Medium (DMEM), resazurin, propidium iodide (PI) 5,5′,6,6′-tetrachloro-41,1′,3,3′ tetraethylbenzimi-dazolylcarbocyanine iodide 100 (JC-1), 2′,7′-dichlorodihydrofluorescein diacetate (DCFH-DA), Annexin-FITC/propidium iodide staining kit were purchased from Sigma Aldrich, USA. Fetal Bovine Serum (FBS) was purchased from GIBCO-BRL. All other chemicals and reagents were of analytical grade.

### Fungal material

The fungus *Chaetomium globosum* PG 1.6 (accession number MH645800) was isolated in our previous study from a marine green alga *C. media* collected from the Konkan coast, India^18^. The fungus was maintained on Potato Dextrose agar supplemented with 250 mg/mL of streptomycin and artificial sea salts^18^.

### Fermentation and extraction of cytotoxic secondary metabolites

*Chaetomium globosum* PG 1.6 was maintained and cultured following the protocols of Kamat et al.^18^ and Kumari et al.^32^. Briefly, the fungus was precultured for seven days on PDA. Erlenmeyer flasks containing 200 mL of PDB were then inoculated with three mycelial agar discs and incubated in static conditions for 28 days at 28 ± 2 °C. Further, the mycelium and fermented broth were separated. The mycelium was dried for 24 h, crushed and added back into the fermented broth. Extraction was performed with 400 mL of 100% ethyl acetate for 24 h after which the organic phase was collected and evaporated to dryness under reduced pressure using a rotary evaporator to obtain a total culture crude ethyl acetate extract.

### Maintenance of cell lines

Human cell lines including HeLa, A549, MCF-7, A431, and HEK 293 T were maintained in complete DMEM supplemented with 10% inactivated FBS and 2 mM glutamine. To avoid contamination, the medium was supplemented with 100 mg/L penicillin and 250 mg/L streptomycin. The cells were grown at 37 °C for 24 h in 5% CO_2_ and 95% humidity in a CO_2_ incubator^18^. These cell lines were employed to determine in vitro cytotoxicity and mechanistic studies of the crude extract and the isolated compound.

### In vitro cytotoxic activity analyses of *C. globosum* total culture ethyl acetate extract by MTT and resazurin reduction assay

The extract was evaluated for its cytotoxic potential by MTT and Resazurin reduction assay on four cancer cell lines (HeLa, A549, MCF-7, and A431) and HEK 293 T cells following the protocol of Kamat et al.^18^.

The respective cell lines were seeded in a transparent, flat bottom 96 well plate at a density of 1 × 10^4^ cells/mL and maintained as per the above-mentioned conditions. After 24 h, the cells were treated with different concentrations of the crude extract respectively and incubated for 48 h at 37 °C in a CO_2_ incubator. For MTT assay, 10 μL of 5 mg/mL MTT solution prepared in PBS was added to each well and incubated for 2 h in a CO_2_ incubator. Further, the plates were emptied and 100 μL of DMSO was added in each well to dissolve the purple MTT-formazan crystals. Absorbance was recorded in a 96 well plate reader Infinite M2000 PRO (Tecan, Crailsheim, Germany) at 595 nm. 0.1% of DMSO treated cells were used as negative control. For resazurin reduction assay, after 48 h of treatment, 20μL resazurin solution (0.01% w/v in double-distilled water) was added to each well. The plates were incubated for 3 h at 37 °C in dark conditions. Fluorescence was recorded on Infinite M2000 PRO plate reader (Tecan, Crailsheim, Germany) using an excitation wavelength of 544 nm and an emission wavelength of 590 nm. The IC_50_ values were calculated for each cell line for both the assays. All the assays were performed in triplicates and results are presented as mean ± SD^46^.

### Standardization of growth conditions and cytotoxic secondary metabolite extraction from *C. globosum* PG 1.6

To study the production profile of cytotoxic metabolites and the growth curve of *C. globosum* PG 1.6, the fungus was grown in PDB for 35 days. After every seven-day interval, extraction was performed of three flasks. This was done for 35 days. Ethyl acetate was used as the extraction solvent to determine the optimal incubation time for the production of cytotoxic secondary metabolites. All the extracts were evaluated by MTT assay on MCF-7 and A549 cells at various concentrations ranging from 3 to 25 µg/mL. All the other conditions were maintained as previously described. Mycelial dry weight was calculated based on which the growth curve was plotted. The incubation period that demonstrated the maximum cytotoxicity was fixed for the subsequent experiments^18,32^.

### Optimization of organic solvent for extraction of cytotoxic secondary metabolites

To investigate the influence of the extraction solvent, the fungus was grown for the optimized incubation period as determined in the above-mentioned study. Total culture extraction was performed with four different solvents of different polarities (hexane, dichloromethane, chloroform, and ethyl acetate). As described earlier, all the extracts were evaluated by MTT assay on MCF-7 and A549 cells at various concentrations ranging from 3 to 25 µg/mL. The solvent that demonstrated the best cytotoxicity was fixed for the subsequent experiments^18,32^.

### Gas chromatography-mass spectroscopy (GC–MS) analyses of *C. globosum* ethyl acetate extract (CGEE)

Phytochemical analyses of biologically active *C. globosum* ethyl acetate extract was carried out using GC–MS. The crude extract was derivatized by silylation to confer volatility for the thorough detection of compounds present in the extract. The derivatized sample was diluted ten times and 1µL of it was subjected to GC–MS analyses. The identification of myco-components was accomplished using the NSIT database^18,47^.

### Purification of chrysin from *C. globosum* ethyl acetate extract

Thin Layer Chromatography (TLC) was performed using silica gel 60 GF_254_ TLC sheets (Merck) 20 × 20, 0.25 mm thickness. The separation of ethyl acetate crude extract for purification of chrysin was optimized using nine different solvent systems (ST1)^48^. 1 µg/mL of standard chrysin (SChr) from (Sigma-Aldrich) dissolved in methanol was also run simultaneously on the same silica gel sheet. The solvent was allowed to run 2 cm below the top of the sheet after which it was removed and dried. It was then subjected to UV 254 nm light. Solvent system No. 3 (toluene:ethyl acetate:acetic acid, 36:12:5) demonstrated the best separation of CGEE and standard chrysin. Hence, it was used to perform preparative TLC. The CGEE TLC band corresponding to the SChr band was scrapped, dissolved in methanol, and centrifuged at 1,000 rpm for 10 min. The supernatant was filtered through a 0.2 µ filter, concentrated under vacuum, and used for subsequent experiments^49^.

### Identification of purified TLC band

#### GC–MS of CGEE TLC band corresponding to SChr

The TLC band was identified using GC–MS without derivatization as mentioned earlier. The compound was identified by the mass spectral database search of the National Institute of Standards and Technology (NIST) followed by matching the mass spectral data^50^.

### UV–Vis spectrophotometry

The UV–Visible spectra of the CGEE TLC band corresponding to SChr and SChr dissolved in methanol were analyzed by UV spectrometry in a 1.0 cm quartz cuvette between 200 and 800 nm in Perkin Elmer Lambda-35 UV/Vis spectrophotometer at a resolution of 2 nm^51^.

### High-performance liquid chromatography analysis

The purity of the isolated compound corresponding to standard chrysin on TLC was further determined by HPLC. The sample, standard chrysin, and CGEE were dissolved in HPLC grade methanol and filtered through a 0.45 µm filter respectively. Chromatographic separation was accomplished using a C18 column equipped on Agilent compact 1120 system. The separation was performed using mobile phases 50% methanol (A) and 50% acetonitrile/water (B) at a flow rate of 1.0 mL/min and detected at 310 nm^52^.

### Liquid chromatography-mass spectrometry (LC–MS) analysis

In order to confirm the presence of chrysin in CGEE, the crude extract was subjected to LC–MS analysis along with the purified compound and SChr^53^. The samples were dissolved in HPLC grade methanol and were analyzed on Bruker Impact HD ESI-QTOF coupled with DIONEX Ultimate 3,000 micro-LC system. The source parameters were as follows: Endplate offset 500 V, Capillary 4,500 V, Nebulizer 60.0 psi, dry gas 120.0 L/min, dry temperature 220 °C, MS, MS/MS performed in negative mode. Acquisition parameter: Mass scan range 50–1,700 m/z. LC parameters: Column-Agilent poroshell 120 (4.6 × 150 mm) SB-C18 2.7 µm particle size, column temperature 25 °C. Gradient elution was performed with mobile phases A (water with 0.1% acetic acid) and B (acetonitrile) where B was maintained at 30% from 0 to 0.5 min, 40% from 0.5 to 10 min, 50% from 10–20 min, 70% from 20–30 min, 90% from 30- 44.9 min and 30% from 44.9 to 45 min^53^.

The MS/MS spectra were analyzed using Metfrag (https://msbi.ipb-halle.de/MetFragBeta/) and Metlin Tandem MS/MS (https://metlin.scripps.edu/) databases.

### Fourier transformed infrared spectroscopy

The solid phase pellets of the CGEE TLC band corresponding to SChr and SChr were subjected to FTIR spectroscopic analysis in the Thermofisher Scientific IR spectrometer. The spectra were recorded and compared in the region of 400–4,000 cm^−1^ with a resolution of 4 cm^−1^ KBr pellet method was followed for sample preparation^54^.

### NMR spectroscopy

The purified fungal chrysin and standard chrysin were dissolved in DMSO-d6 and 1H and 13C NMR analysis were carried out using a Bruker AVANCE 400 MHz spectrometer. NMR data were analyzed using the MestreNova software^54^.

### Evaluation of the anti-cancer activity of purified chrysin

#### In vitro cytotoxicity analyses by MTT assay

The purified fungal chrysin (FChr) and SChr were subjected to MTT assay as described earlier, to check for their in vitro cytotoxic activity on MCF-7 and HEK 293 T cells for 72 h at different concentrations (20 to 250 µM) while 0.1% DMSO treated cells were used as negative control^18,55^.

The selectivity index (SI) for fungal and standard chrysin was calculated using the formula^42^: SI = IC_50_ of the compound on non-malignant cell line/IC_50_ of the compound on the malignant cell line.

#### In vitro cytotoxicity analyses of CGEE, purified chrysin and standard chrysin by PI live/dead cell assay

MCF-7 cells were seeded at a density of 3 × 10^5^ (1,000 µL/well) in a 24 well plate and maintained in standard conditions. After 24 h, CGEE treatment (3, 5, 10, 25 µg/mL) was given for 24 h. Treatment of FChr and SChr was also given at concentrations ranging from 5 to 250 µM for 72 h. After the treatment, cells were trypsinized and washed twice with PBS. The cells were resuspended in 200 µL of PBS. The stain PI (1 µg/mL) was added just before acquiring data. Analyses were done in the CytoFlex flow cytometer (Beckman Coulter) by CytExpert 2.0 software^18^.

### Cell cycle analyses

Cell cycle phase arrest and distribution were evaluated using PI staining followed by flow cytometry analysis as described by Posadino et al.^56^. Briefly, 2 × 10^5^ cells were seeded in a 6 well plate and incubated for 24 h in standard conditions to form a monolayer. After treatment of CGEE, FChr, and SChr, the adherent cells were trypsinized, washed twice with PBS, fixed with 70% ice-cold ethanol overnight, and finally incubated with RNase A (100 µg/mL) for 5 h. Further, the cells were stained with PI (50 µg/mL) for 30 min at 37 °C in a CO_2_ incubator. The data was acquired in the CytoFlex flow cytometer (Beckman Coulter) by CytExpert 2.0 software for a minimum of 10,000 cells per sample. 100 nM Paclitaxel treated cells served as positive control while cells treated with 0.1% DMSO served as a negative control.

### Mitochondrial membrane potential assay

Mitochondrial Membrane Potential (MMP) loss was measured by flow cytometry using a JC-1 probe as described by Kamat et al.^18^ and Posadino et al.^56^. Briefly, cells were seeded in a 24 well plate at a density of 3 × 10^5^ cells/well. After 24 h cells were treated with various concentrations of CGEE, FChr, and SChr. As described earlier, after treatment intervals, cells were trypsinized and washed twice with PBS. The cells were further stained with freshly prepared JC-1 (2.5 mg/mL) and incubated at 37 °C in dark for 15 min. Subsequently, data were acquired in the CytoFlex flow cytometer (Beckman Coulter) by CytExpert 2.0 software. Carbonyl cyanide 3-chlorophenylhydrazone (CCCP) treated cells served as positive control while 0.1% of DMSO treated cells were used as a negative control.

### Reactive oxygen species production assay

Reactive Oxygen Species (ROS) production within cells was evaluated using the DCFH-DA assay. Briefly, cells were cultured in a 24 well plate at a density of 2 × 10^4^ cells/well. After treatment with the indicated concentrations of CGEE, FChr, and SChr for specific intervals, the cell culture medium was replaced with freshly prepared 10 µM DCFH-DA in DMEM and incubated at 37 °C for 15 min in dark. Further, the cells were trypsinized, washed twice with PBS, and analyzed for the percentage of ROS generation in CytoFlex flow cytometer (Beckman Coulter) by CytExpert 2.0 software. A 15 min treatment of cells with 10 mM H_2_O_2_ prepared in DMEM was used as positive control while 0.1% of DMSO treated cells served as negative control^18,56^.

### Annexin V-FITC/PI assay for the extent of phosphatidylserine exposure

Cells were seeded in a 24 well plate a density of 4 × 10^5^ and incubated for 24 h in standard conditions to form a monolayer. Further, cells were treated with various concentrations of FChr for a specific interval after which the cells were harvested by trypsinization as described earlier. According to the manufacturer’s instructions, the cells stained with 2 µL of the AnnexinV-FITC with or without PI in along with annexin binding buffer. These cells were incubated for 15 min in dark and were then analyzed using CytoFlex flow cytometer and CytExpert 2.0 software. 100 nM Paclitaxel treated cells served as positive control while cells treated with 0.1% DMSO served as negative control^26^.

### Statistical analysis

All the experiments were performed in triplicate and quantitative variables are represented in terms of mean ± S.D. in histograms. For statistical significance, means ± SD of all groups were compared and analysis of variance (ANOVA) was performed using a statistical package, SPSS 16.0 (SPSS Inc., Chicago, IL, USA). The probability of a P-value of ≤ 0.05 was taken to indicate statistical significance. Further, Duncan’s Multiple Range Test (DMRT) was used to identify the pairs of groups where the means are significantly different at α = 0.05^32^.

## Supplementary information

Supplementary Data File 1

Supplementary Data File 2
