# Supplementary material for: Endophytic fungus, Chaetomium globosum, associated with marine green alga, a new source of Chrysin
Source: Sci Rep. 2020 Oct 30;10:18726. doi: 10.1038/s41598-020-72497-3 (PMC7603332; doi:10.1038/s41598-020-72497-3)
Supplement: Supplementary file 1 — Supplementary Data File 1 [file 41598_2020_72497_MOESM1_ESM.docx]

**Endophytic fungus, *Chaetomium globosum,* associated with marine green alga, a new source of Chrysin**

Siya Kamat, Madhuree Kumari, **Kuttuvan Valappil Sajna** and C. Jayabaskaran*

*Department of Biochemistry, Indian Institute of Science, Bangalore-560012, India*

***Corresponding author:**

Prof. C. Jayabaskaran,

Department of Biochemistry,

Indian Institute of Science, Bangalore-560012,

India,

Tel: +91-80-22932482; Fax: +91-80-23600814;

E-mail: [cjb@iisc.ac.in](mailto:cjb@iisc.ac.in)

**Supplementary Table 1 (ST1): List of TLC solvent systems used to optimize the best separation of fungal chrysin**

| **Chromatographic system No.** | **Solvents, ratio** |
| --- | --- |
| 1 | toluene:ethyl acetate:formic acid, 36:12:5 |
| 2 | cyclohexane:ethyl acetate:formic acid, 30:15:5 |
| 3 | toluene:ethyl acetate:acetic acid, 36:12:5 |
| 4 | cyclohexane:ethyl acetate:acetic acid, 31:14:5 |
| 5 | n-hexane:ethyl acetate:formic acid, 31:14:5 |
| 6 | toluene:acetone:formic acid, 38:10:5 |
| 7 | n-hexane:ethyl acetate:acetic acid, 31:14:5 |
| 8 | petroleum ether:ethyl acetate:formic acid, 30:15:5 |
| 9 | carbon tetrachloride:acetone:formic acid, 35:10:5 |


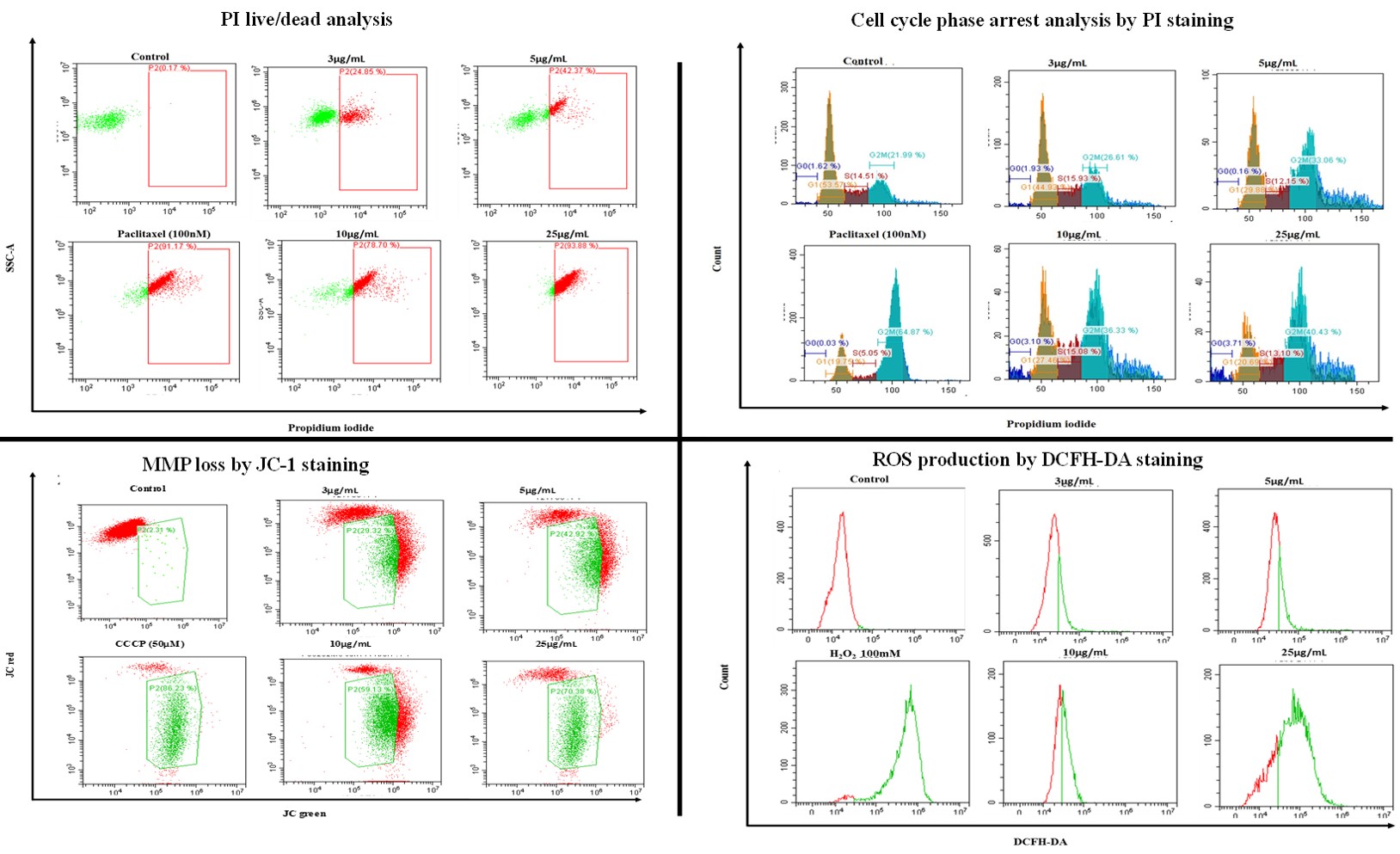


**Supplementary Figure 1 (S1):** FACS profiles of MCF-7 cells treated with CGEE after 24 h analyzed by PI live/dead assay, distribution of cells in cell cycle phases, loss of MMP and ROS level. Flow cytometry data were quantified using the CytExpert 2.0 software.


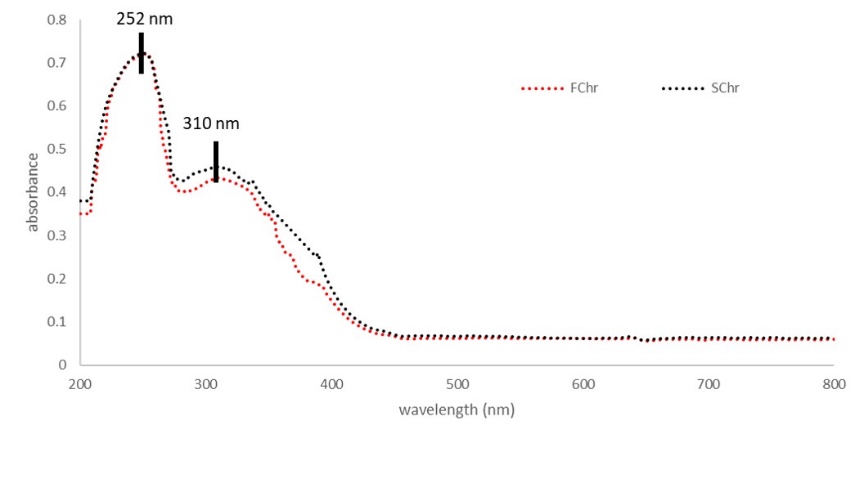


**Supplementary Figure 2 (S2):** Comparative analysis of UV spectrum of purified compound and SChr. Λmax of 252 nm and 310 nm was observed for both the samples


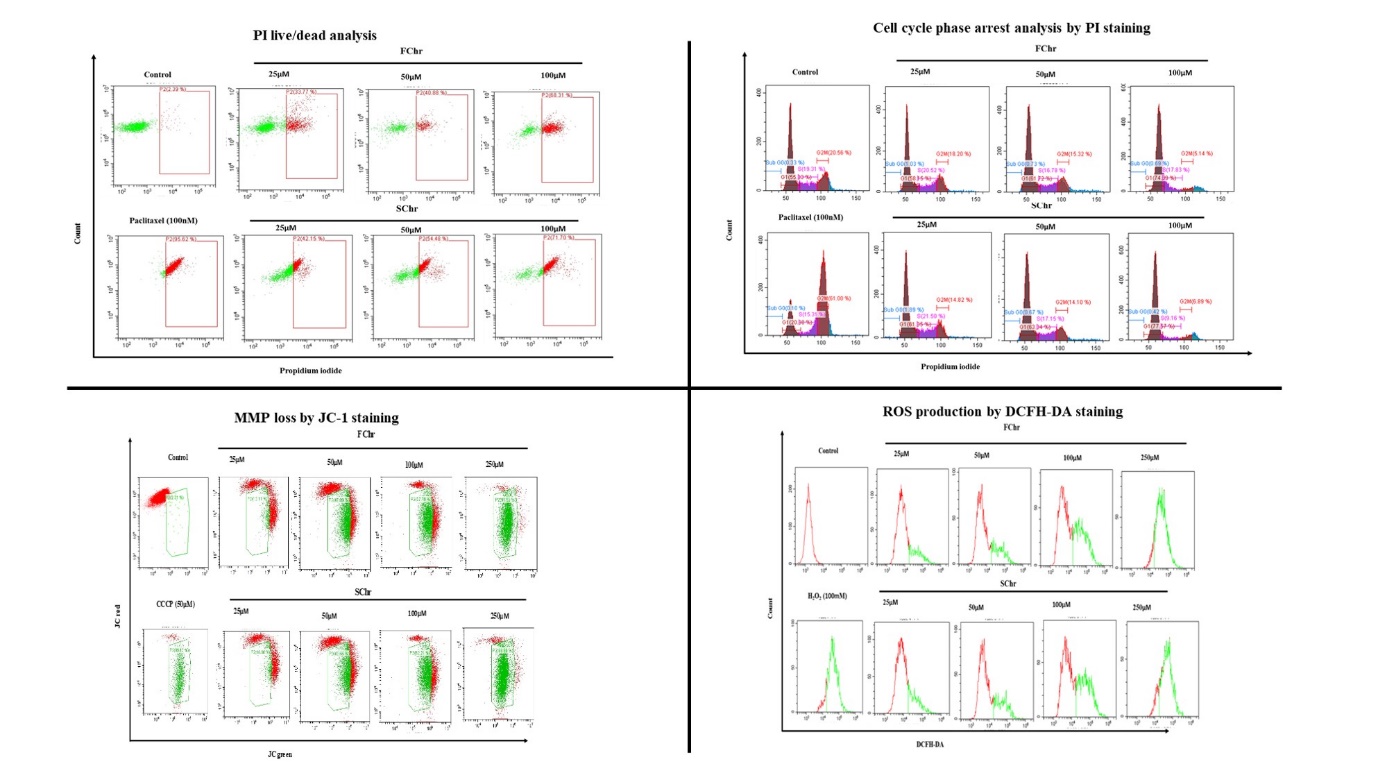


**Supplementary Figure 3 (S3):** FACS profiles of MCF-7 cells treated with 72 h of FChr and SChr analyzed by PI live/dead assay, distribution of cells in cell cycle phases, loss of MMP and ROS level. Flow cytometry data were quantified using the CytExpert 2.0 software.
