# Supplementary material for: Endophytic fungus, Chaetomium globosum, associated with marine green alga, a new source of Chrysin
Source: Sci Rep. 2020 Oct 30;10:18726. doi: 10.1038/s41598-020-72497-3 (PMC7603332; doi:10.1038/s41598-020-72497-3)
Supplement: Supplementary file 2 — Supplementary Data File 2 [file 41598_2020_72497_MOESM2_ESM.docx]

**Endophytic fungus, *Chaetomium globosum,* associated with marine green alga, a new source of Chrysin**

Siya Kamat, Madhuree Kumari, **Kuttuvan Valappil Sajna** and C. Jayabaskaran*

*Department of Biochemistry, Indian Institute of Science, Bangalore-560012, India*

***Corresponding author:**

Prof. C. Jayabaskaran,

Department of Biochemistry,

Indian Institute of Science, Bangalore-560012,

India,

Tel: +91-80-22932482; Fax: +91-80-23600814;

E-mail: [cjb@iisc.ac.in](mailto:cjb@iisc.ac.in)

**
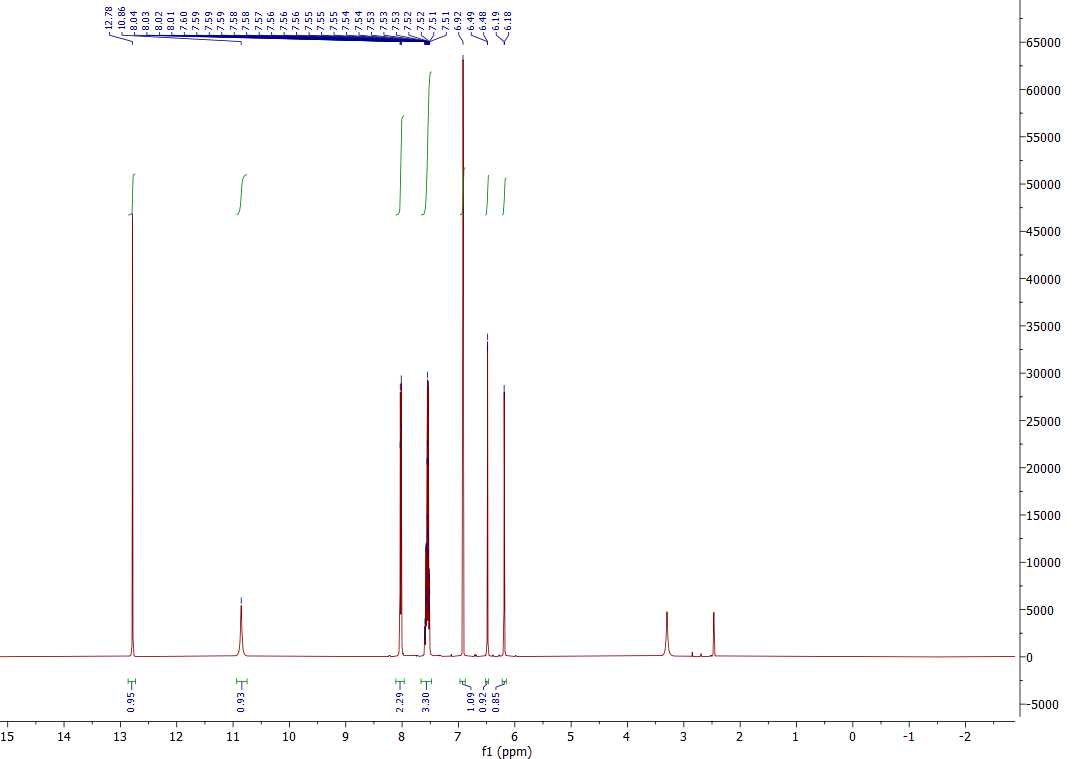
**

**A**

**
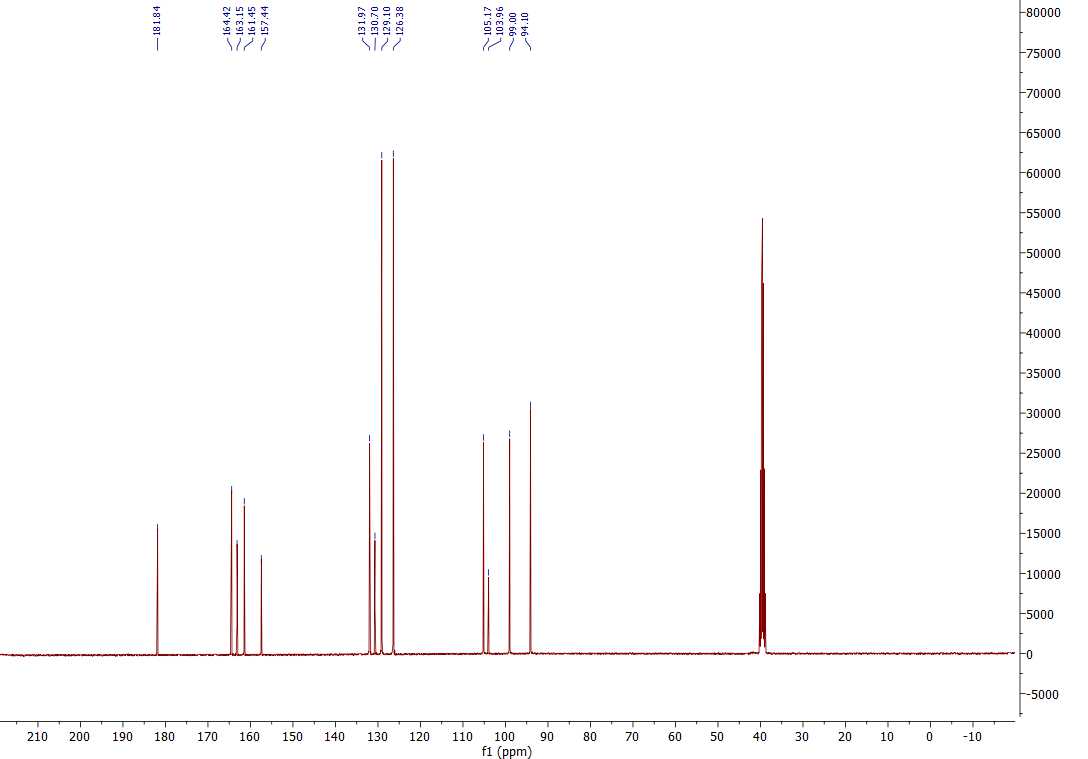
**

**B**

Supplementary Figure. ^1^H NMR (A) and ^13^C NMR (B) spectra of standard chrysin

SCHR:^1^H NMR (400 MHz, DMSO -*d*6), δ (ppm), 12.78 (1H, s, OH-5), 10.85 (1H, s, OH-7), 8.03 (2H, dd, *J* = 7.9 Hz and 1.7 Hz, H-2', 6'), 7.55 (3H, m, H-3', 4', 5'), 6.92 (1H, s, H-3), 6.48 (1H, d, *J* = 2.1 Hz, H-8), 6.19 (1H, d, *J* = 2.1 Hz, H-6).

^13^C NMR (101 MHz, DMSO-*d*6), δ (ppm), 163.15 (C-2), 105.17(C-3), 181.84 (C-4), 161.45 (C-5), 99.00 (C-6), 164.42 (C-7), 94.10 (C-8), 157.44 (C-9), 103.96 (C-10), 130.70 (C-1'), 126.38 (C-2', 6'), 129.10 (C-3', 5'), 131.97 (C-4').

**References**

1. Shen CC, Chang YS, Ho LK. Nuclear magnetic resonance studies of 5, 7-dihydroxyflavonoids. *Phytochemistry*. (1993).
2. Chen, L.J., Games, D.E. and Jones, J. Isolation and identification of four flavonoid constituents from the seeds of Oroxylum indicum by high-speed counter-current chromatography. *Journal of Chromatography A*, **988**. 95-105. (2003)
